# Beauty, the feeling

**DOI:** 10.1016/j.actpsy.2021.103365

**Published:** 2021-07-08

**Authors:** Aenne A. Brielmann, Angelica Nuzzo, Denis G. Pelli

**Affiliations:** a Department of Psychology, New York University, New York, NY 10003, USA; b Department of Computational Neuroscience, Max-Planck Institute for Biological Cybernetics, Tübingen, Germany; c The Graduate Center, City University of New York, New York, NY 10016, USA; d Center for Neuroscience, New York University, New York, NY 10003, USA

## Abstract

Many philosophers and psychologists have made claims about what is felt in an experience of beauty. Here, we test how well these claims match the feelings that people report while looking at an image, or listening to music, or recalling a personal experience of beauty. We conducted ten experiments (total *n* = 851) spanning three nations (US, UK, and India). Across nations and modalities, top-rated beauty experiences are strongly characterized by six dimensions: intense pleasure, an impression of universality, the wish to continue the experience, exceeding expectation, perceived harmony in variety, and meaningfulness. Other frequently proposed beauty characteristics — like surprise, desire to understand, and mind wandering — are uncorrelated with feeling beauty. A typical remembered beautiful experience was active and social like a family holiday — hardly ever mentioning beauty — and only rarely mentioned art, unlike the academic emphasis, in aesthetics, on solitary viewing of art. Our survey aligns well with Kant and the psychological theories that emphasize pleasure, and reject theories that emphasize information seeking.

## 1. Introduction

Beauty has fascinated humankind for millennia. Most of the great philosophers tried to define it, including Plato (428–348 BCE; Denham, 2012), Aristotle (384–322 BCE; Halliwell, 1986), Kant (1724–1804; Nuzzo, 2005), and Hegel (1770–1831; Nuzzo, 2006). The pioneer of modern psychology, Gustav Fechner (1801—1887), took great interest in empirically testing what beauty is (Fechner, 1876). Yet, in contemporary models of aesthetic experience, beauty is just one of many undefined aesthetic responses (e.g., Leder & Nadal, 2014; Pelowski et al., 2017).

### 1.1. Philosophical accounts of beauty

The philosophical theories of beauty specify the contributions of many experiential dimensions to the *experience* of beauty. These elaborate philosophical theories aim to paint a complete picture of all the variables associated with intense beauty experiences. First and foremost, except for Hegel, they all relate beauty to pleasure (Nuzzo, 2006). Hume (1739, section VIII) in particular stressed the importance of “Pleasure and pain…” as “…not only necessary attendants of beauty and deformity, but … their very essence.” With the exception of Hegel, most philosophers agree that experiencing beauty involves the experience of pleasure. They do, however, add additional requirements that an experience of pleasure needs to fulfill in order to qualify as an experience of beauty. Philosophers like Plato (Denham, 2012) and Kant (Nuzzo, 2005) observe that one’s own beauty experiences seem universally valid. This subjectively perceived universality means, as Kant argues, that even though you may know that someone else does not share your opinion on the beauty of a particular object, you perceive their differing opinion as wrong and your own opinion as right. What is beautiful to you *ought* to be beautiful to others, too. In contrast to Kant and Plato, Hume (Taylor, 2008) and Santayana (1955) do not claim that beauty is necessarily perceived as universal. Another experiential dimension that differentiates philosophers’ conceptions of beauty is surprise. Hegel (Nuzzo, 2006) and Aristotle (Halliwell, 1986) viewed surprise as a central element of experiencing beauty, whereas it played little role for others (like Kant, Plato, and Hume). Santayana even went so far as to claim that surprise is a measure of ugliness, and thus detrimental to beauty (Santayana, 1896, §29).

A complete discussion of each philosopher’s perspective is beyond the scope of this empirical article, but it is worth noting that many experiential dimensions, including pleasure and surprise, appear in all the philosophical theories. We exploit this consistency here and systematically score how well each theory matches contemporary feelings of beauty (see Table 2).

### 1.2. Psychological accounts of beauty

In contrast to their philosophical cousins, psychological theories of beauty each typically consider only the few dimensions that it supposes to be essential to experiencing beauty. These brief theories are parsimonious in order to predict beauty with just a few predictors that are not strongly correlated with each other.

To date, the psychological literature on beauty has focused on identifying object properties or contexts that influence aesthetic evaluations in general, of which beauty is one (see Brielmann & Pelli, 2018, for a review). The study of human beauty – in terms of facial attractiveness – has amassed the most comprehensive data in this regard (Rhodes, 2006), followed by other visual objects (Palmer et al., 2013). People, on average, find symmetric, round, and familiar objects most (visually) appealing. Scholars of music science have, in parallel, studied the properties of aesthetically pleasing music and seem to converge on a theory that states that music is most appealing when it is familiar yet surprising (e.g., Koelsch et al., 2019). An exhaustive review of the particular object properties that, in each respective domain, is related to beauty *judgments* is beyond the scope of this article. Instead, we want to highlight and test theories about what constitutes the *experience* of beauty, independent of the properties of the object that caused this experience. In the following, we will briefly present the main components of the most prominent instances of the various theories on beauty as an experience. In the interest of brevity, we refer the interested reader to the original publications for a detailed description of the underlying theories.

Fechner (1876) defined beauty as an experience of pleasure (see Brielmann, 2020, for an English translation). He explicitly conceptualized beauty in its broadest sense as anything that immediately elicits pleasure. Much as we do here, he based this broadest definition of beauty on people’s use of the terms “beauty” and “beautiful” in daily life. Berlyne (1971) saw aesthetic appreciation, i.e., beauty, as one outcome of a more general search for optimal hedonic value, and thus optimal potential arousal. Even though he initially considered many “collative” variables (i.e., novelty, surprisingness, incongruity, complexity, variability, and puzzlingness) that determine this arousal potential, he and subsequent research found that these ultimately collapse to three factors: hedonic tone (pleasure), arousal (excitement), and simplicity (Berlyne, 1974; see also Marin et al., 2016). Leder and Nadal (2014) place the meaningfulness of an experience at the heart of beauty. In their model, aesthetic judgments like beauty are the outcome of a successive evaluation that culminates in cognitive mastery. Crucially, when an observer determines that the object in question is meaningless, this model predicts that it will be evaluated negatively, i.e., as not beautiful. A growing new perspective in music science posits that passages of music are most liked, or beautiful, when the pleasure they elicit exceeds the listener’s expectation (Salimpoor et al., 2015).

Some psychological approaches towards beauty identify crucial components (or determinants) of beauty and outright equate beauty with other measurable feelings. Vessel et al. (2013) identify the state of being moved as equivalent to what others call “beauty”. Diessner et al. (2018) equate beauty with unity in variety, i.e., a feeling that the various elements of an object are harmoniously combined into a coherent whole. (This is similar to Wittgenstein’s (1938) description of “clicking or fitting” or “harmony” in aesthetic judgement.)

While these models focus on the relation between beauty and the perceiver’s subjective feelings, others emphasize the relation between beauty and information-seeking, broadly construed as learning, interest, or wanting to understand the experience. Some theories attribute experiences of beauty—broadly construed as pleasure derived from sensory experiences—to gratification of a basic need to improve one’s knowledge of the sensory environment (Biederman & Vessel, 2006; Brielmann & Dayan, 2021). These drive-based theories do not claim that the observer is aware of the information gain associated with beauty, but several other such theories do. Armstrong and Detweiler-Bedell (2008) proposed that beauty is not only pleasurable but crucially contains an element of learning. Specifically, they propose that beauty is an emotion that is linked to progress with regard to “the mind’s abstract, epistemic goals”, and it is therefore said to be elicited by challenging, complex stimuli that have a potential for being understood better over time. Reber et al. (2004) suggested that beauty lies in the ease of processing during the experience, i.e., a feeling that understanding is attained. Thus, much like Armstrong and Detweiler-Bedell, a feeling about understanding is paramount to their definition of beauty, but in Reber and colleagues’ view, rather than the feeling of *progress towards*, it is instead a feeling of having *achieved* understanding that correlates with beauty: understanding, not learning. Within the realm of music, Kivy (1990) claims that a piece of music must be interesting in order to be beautiful.

The great variety of proposed dimensions has yet to be winnowed by the contemporary field of empirical aesthetics to discover which dimensions, alone or in combination, are generally characteristic of beauty experiences across object kinds. To date, we lack a data-driven definition of what it means to experience beauty.

### 1.3. Overview of the current studies

We conducted ten studies in which we asked more than 800 participants to rate several dimensions, including beauty, of various experiences. Psychological theories aim to predict beauty ratings, so we asked each participant to rate images and music of various degrees of beauty. Philosophical theories aim to discover correlates of beauty, so we asked each participant to rate a remembered, intense, beauty experience. Together, these results reveal six experiential dimensions that characterize intense beauty.

To cast our net widely, we first assessed eleven of the dimensions that have been considered by prominent philosophers of aesthetics: 1) pleasure, 2) wishing to continue the experience, 3) feeling alive, 4) feeling that the experience is beautiful to everyone, 5) number of felt connections to the experience, 6) longing, 7) feeling free of desire, 8) mind wandering, 9) surprise, 10) wanting to understand the experience more, and 11) feeling that the experience tells a story. Each of the seven included philosophers made statements about each of these dimensions, so we could assess whose definition best fit the empirical data.

Second, we measured people’s responses to eight additional dimensions brought forward by psychologists: 1) complexity, 2) arousal or excitement, 3) learning from the experience, 4) wanting to understand, 5) harmony in variety, 6) meaningfulness, 7) exceeding one’s expectation, and 8) interest. (Our Supplementary Material specifies the exact wording of each question and cites the authority who inspired it.)

In addition, we also asked our participants about their explicit beliefs about beauty at the end of each experiment to probe their endorsement of seven more beauty dimensions.

While seeing, hearing, or remembering beauty, our participants rated the beauty and many other dimensions inspired by either philosophy or psychology. In this way, we identify the general characteristics of intense beauty experience across modalities. We here focus on a descriptive approach that allows us to identify experiential dimensions associated with intense beauty. By uncovering which dimensions are consistently correlated with intense beauty, we provide the first data-driven definition of the beauty experience.

## 2. Methods

For each experiment, we recruited 100 or more participants via Amazon Mechanical Turk (MTurk). We chose to recruit participants through MTurk, and in three (English-speaking) countries, rather than from the participant pool available at New York University because our study aimed to sample a population that is more representative of the world population. Online data collection also allowed us to recruit similarly in all three countries. Inclusion criteria were: MTurk workers with an approval rate of at least 90%, who have completed at least 50 HITs, and reside in the country targeted in the particular experiment. These inclusion criteria are commonly used to assure that only such participants are recruited who have demonstrated that they reliably perform the task they have been assigned (e.g., Brielmann & Pelli, 2019; Kurdi et al., 2017). In addition, most of the experiments reported below required that participants provide a written response to an open-ended question, allowing us to screen participants for failure to follow the instructions of the question based on their answers.

All participants consented to participate according to a consent form approved by the NYU UCAIHS (university committee on activities involving human subjects; IRB-FY2016–404) by checking a box in the online form. Participants were reimbursed $15 per hour. The payment depended on the duration of the experiment. All data were collected between April 2019 and May 2020. Raw data and analysis scripts are openly accessible at https://github.com/aenneb/characterizing_beauty.

### 2.1. General methods

#### 2.1.1. Procedures

Participants rated images (Experiment 1a), images and music (Experiment 1b), or memories (Experiment 2–4) on the same 12 dimensions: beauty, pleasure, surprise, wanting to experience the stimulus longer, feeling free of desire, feeling alive, wanting to understand the experience more, mind wandering, number of connections felt with the experience, how far the experience tells a story, how far the experience is beautiful (or provides relief in Experiment 4) for everyone, and longing. Participants in Experiments 2–4 rated experiences on a further 2 dimensions. In Experiment 2b, an additional set of four questions was added as well as a question about whether they had the experience alone. All questions are listed in the Supplementary Material. All ratings were given on a scale from scale from “not at all” (1) to “very much” (7), except for the scale asking about the number of connections with the experience (“None” (1) to “Many” (7)). At the end of each experiment, participants also answered six questions about their general beliefs about beauty, and their age and gender. Participants in Experiments 1a and 2b also provided additional demographic information. These additional questions were removed from other experiments because none of these additional variables correlated with the ratings.

#### 2.1.2. Analyses

After initial inspection, the data were analyzed with R version 3.5.3, python version 3.6.8, and MATLAB version R2018b.

### 2.2. Experiment 1a: rating images of various degrees of beauty (USA)

#### 2.2.1. Participants

Of the 100 recruited participants, 99 completed the survey. Of these, 66 were male, 31 female, and 2 did not disclose their gender. Their ages ranged from 20 to 72 with a mean age of 34.8 (*SD* = 9.9). Most had earned a college degree (*n* = 50), or at least some college education (*n* = 24). The remaining participants either had a high school (*n* = 17) or graduate degree (*n* = 8). Most participants had neither any formal art (*n =* 83) nor any philosophy education (*n* = 87). A few attended some art (*n* = 13), or philosophy courses (*n* = 11). Only three participants had a degree in art or art history, and only one had a degree in philosophy. Most participants’ household income ranged between $50,000 and $70,000 (*n* = 42), with 17 falling below that range, and 40 above. Most (*n* = 73) participants were white, seven identified as American, seven as Indian, seven as multiracial, five identified as black, five as Hispanic, one identified as South Asian, and one as other. On average, participants identified as more liberal than conservative on a 1 (very liberal) to 8 (very conservative) scale, *M* = 3.3, *SD* = 1.9.

#### 2.2.2. Stimuli

We selected the five most beautiful image from the open affective standardized image set (OASIS; Kurdi et al., 2017) based on a previous study (Brielmann & Pelli, 2019), as well as three images with median beauty ratings. (Beauty ratings differ widely across participants. “Most beautiful” above refers to the images with the highest average ratings.) The OASIS consists of a diverse set of stock images. We will therefore refer to these stimuli as *beautiful stock-images (B)* and *neutral stock-images (N),* respectively. In addition, we selected the five most beautiful art images of a diverse set of paintings previously used by Belfi and colleagues (2019). We will refer to these images as *beautiful art-images*.

#### 2.2.3. Procedures

On each trial, the participant saw one image with a rating scale below it. The image and rating scale were displayed until the participant did the rating and clicked the “next” button. Thus, each trial yielded one rating for one stimulus. Four beautiful art- and stock-images and two neutral stock-images were rated on each of 12 dimensions. One image of each category was rated twice on each dimension, the remaining stimuli were rated once. The order of images and ratings was randomized for each participant.

#### 2.2.4. Analyses

For all analyses involving demographics, we binarized the art and philosophy education demographic into “has” (some courses or degree) or “has not”. We fit linear mixed-effects models with the R package *lme4* (Bates et al., 2015) and obtained further statistics with the *lmerTest* (Kuznetsova et al., 2017) and *MuMIn* packages (Bartoń, 2019). The linear models predicted beauty ratings. We successively evaluated linear mixed-effects models, starting with the simplest (only including random effects of either stimulus or participant), then adding fixed effects of all eleven remaining ratings, and lastly exploring the interaction of demographic variables with the linear combination of the eleven ratings. The best model was selected based on the Bayesian Information Criterion (BIC) calculated on the overall fit as well as average BIC after 10-fold cross-validation. Since the Likert scale ratings we collected cannot be assumed to be interval scaled, we further confirmed the robustness of our findings with Cumulative Link Mixed Models (CLMM) that treated all ratings as ordinal variables using the *clmm* function of the R package *ordinal*. Differences between continuous variables were tested with two-tailed *t*-tests, those between ordinal variables with Wilcoxon-rank-sum tests and those between proportions with the built-in *prop.test* function in R. We used MATLAB R2018b to run cluster analyses.

### 2.3. Experiment 1b: rating beautiful images and music. (USA)

#### 2.3.1. Participants

Of the 100 recruited participants, 99 completed the survey. We excluded an additional six participants due to ≥5% wrong responses on the question asking about the stimulus type (see Procedures below). Thus, we analyzed data from 93 participants. Of these, 51 were male, and 42 female. Their ages ranged from 18 to 64 with a mean age of 36.8 (*SD* = 10.8).

#### 2.3.2. Stimuli

Participants saw the same five most-beautiful images from the OASIS (Kurdi et al., 2017) as used in Experiment 1a. In addition, they listened to the “greatest-hot-100-singles of all time” according to the music billboard charts (https://www.billboard.com/charts/greatest-hot-100-singles). Both images and music were chosen so as to maximize the beauty rating.

#### 2.3.3. Procedures

The procedures were identical to Experiment 1a except for the changes necessary to ensure that participants listened to the music. To do so, the main experiment was preceded by explicit instructions to turn on speakers or headphones and a sound check question. In 50% of the trials in this experiment, we played the beginning of one the five songs. After each trial, we asked participants whether they saw an image or listened to a song with an additional open-ended “other” option. Trials in which “other” was selected were excluded from the analyses.

#### 2.3.4. Analyses

We used the same linear mixed model analyses as in Experiment 1a.

### 2.4. Experiment 2a: rating remembered beauty. (USA)

#### 2.4.1. Participants

Of the 100 recruited participants, 99 completed the survey. Based on the written memory descriptions provided, we excluded seven participants (4 men, 3 women) due to apparent non-compliance, e.g., describing the remembered beauty experience with a single word. Of the remaining 92 participants, 71 were male, 21 female. Their ages ranged from 19 to 70 with a mean of 34.3 years (*SD* = 10.2).

#### 2.4.2. Procedures

We told participants to “Please think back to an experience during which you felt intense beauty. Picture the experience. Remember as many details as you can: what you saw, heard, smelled, and felt. Let the memory linger for a minute.” A timer counted down 1 min in 10-second intervals. Participants were only able to continue the survey after a minimum of 1 min had elapsed. Next, we asked them to provide a written description of their experience. On the next page, we asked them how long ago the experience had occurred, and let them rate the experience on the 12 dimensions listed in General methods plus two dimensions that we added based on further discussion of the results of Experiment 1, namely perfection and peacefulness.

#### 2.4.3. Analyses

We compared ratings for *top-rated* immediate- and remembered-beauty trials (i.e., trials with beauty ratings = 7). For the data of Experiment 1, this means that we included 1 to 18 trials of each of 71 participants, a total of 356 trials. Fifty-three of the 90 participants in Experiment 2 were included. We used the python package *NLTK* (Loper & Bird, 2002) and *empath-client* (https://github.com/Ejhfast/empath-client; Fast et al., 2016) to analyze written memory descriptions.

### 2.5. Experiment 2b: replication and extension of experiment 2a. (USA)

#### 2.5.1. Participants

One hundred and one participants completed the survey. Based on the written memory descriptions provided, we excluded 12 participants due to apparent non-compliance. Of the remaining 89 participants, 57 were male, 32 female. Their ages ranged from 19 to 64 with a mean of 34.9 years (*SD* = 11.0). Similar to our first experiment, we collected more extensive demographic information regarding education as well as religion, which is available in the Supplementary Material.

#### 2.5.2. Procedures & analyses

The main procedures and analyses were identical to Experiment 2a with two exceptions.

One, we added a perceptual judgement of an objective physical property to assess individual response bias. Participants were shown two gray circles for 1 s and asked to rate which one was bigger and by how much. The right circle’s diameter was 90% of the left circle’s. Importantly, the rating was given on the same scale, from “Not at all” (1) to “Very much” (7), that was used to rate subjective properties. With this rating, we assessed variation in general response bias across observers by correlating the circle-size comparison rating with ratings on all other dimensions using Pearson’s correlations.

Two, we included six new questions after reviewing the data from Experiments 3–6: 1) “Did this experience give you a new perspective on other experiences?”, 2) “How strong do you think this experience would be if you had it again?”, 3) “Would sharing this experience with friends make it better?”, 4) “Did this experience give you a new perspective on yourself?”, 5) “Were you alone when you had this experience?”, 6a) if “yes” to 5: “Did you wish you could share this experience with others?”, 6b) if “no” to 6: “Did the experience make you feel more connected with the people you were with?”

### 2.6. Experiment 3a: remembered beauty. (UK)

#### 2.6.1. Participants

We initially aimed to recruit 100 participants from the UK via Amazon Mechanical Turk. Of the 100 recruited participants, 99 completed the survey. Based on the written memory descriptions provided, we excluded 21 participants due to apparent non-compliance such as one-word beauty memory descriptions. Of the remaining 78 participants, 51 were male, 27 female. Their ages ranged from 19 to 70 with a mean of 34.3 years (*SD* = 10.2).

#### 2.6.2. Procedures & analyses

All procedures and analyses were identical to Experiment 2a.

### 2.7. Experiment 3b: remembered beauty. (India)

#### 2.7.1. Participants

Even though we requested only 150 participants, 154 completed the survey. Based on the written memory descriptions provided, we excluded 102 participants due to apparent non-compliance: including several duplicate answers, copy-pasted responses unrelated to the task at hand, and one-word beauty memory descriptions. Of the remaining 52 participants, 43 were male, 9 female. Their ages ranged from 22 to 40 with a mean of 27.9 years (*SD* = 4.4).

#### 2.7.2. Procedures & analyses

The main procedures and analyses were identical to Experiment 2b, with the sole exception that the size of the smaller circle for the perceptual task to was 80% the diameter of the larger one.

### 2.8. Experiment 4: remembered relief. (USA)

#### 2.8.1. Participants

We recruited 100 participants from the USA via Amazon Mechanical Turk. All completed the survey. Based on the written memory descriptions provided, we excluded 10 participants due to apparent non-compliance such as one-word memory descriptions. Of the remaining 90 participants, 57 were male, 33 female. Their ages ranged from 20 to 69 with a mean of 35.8 years (*SD* = 12.1).

#### 2.8.2. Procedures & analyses

All procedures and analyses were identical to Experiment 2a, except that we substituted “relief” for “beauty” in the instructions. To ratings of remembered beauty and relief on 14 dimensions, we first tested whether an overall difference exists with a MANOVA, followed by separate two-sided *t*-tests for each rating.

### 2.9. Experiments 5–6: rating stimuli on dimensions derived from the psychology literature. (USA)

After establishing which dimensions considered by philosophers were correlated with people’s beauty experiences in Experiments 1–2, we turned to contemporary psychology theories of beauty to test which of their suggested features correlate with beauty. To do so, we replicated Experiments 1a, 1b, and 2b with a different set of questions derived from the main psychological theories that make statements about the experience of beauty (see Supplementary Material for details and references). All stimuli and instructions were kept the same; only the questions were changed. We list the most important information about the participants below. More extensive demographic information is available in the Supplementary Material. Analyses for Experiments 5–6 were the same as for the Experiments 1a, 1b, and 2b.

#### 2.9.1. Experiment 5a – rating images

One hundred and one participants completed the survey. Of these, 63 were male, and 38 female. Their ages ranged from 20 to 73 with a mean age of 37.8 (*SD* = 11.9).

#### 2.9.2. Experiment 5b – rating images and music

One hundred participants completed the survey. We excluded fifteen participants due to ≥5% wrong responses on the question asking about the stimulus type (see Experiment 2b above). We thus analyzed data from 85 participants. Of these, 48 were male, and 37 female. Their ages ranged from 22 to 72 with a mean age of 37.8 (*SD* = 11.5).

#### 2.9.3. Experiment 6 – rating memories

Of the 100 recruited participants, 99 completed the survey. Based on the written memory descriptions provided, we excluded 27 participants due to apparent non-compliance such as one-word beauty memory descriptions. Of the remaining 72 participants, 34 were male, 36 female, one identified with another gender, and one preferred not to answer the question. Their ages ranged from 22 to 69 with a mean of 39.5 years (*SD =* 12.5).

## 3. Results

### 3.1. Variables correlated with beauty ratings of images and music

In our stimulus experiments, we analyzed responses by 192 participants in the USA. All together, the participants rated a total of 20 different stimuli: Experiment 1a. 5 beautiful stock-images; 5 beautiful art images; 5 neutral stock-images; Experiment 1b. 5 beautiful stock-images from Experiment 1a; the top 5 billboard musical hits of all time (details in Methods). We used mixed-effects linear models (Bates et al., 2015) to assess which aspects of the experience are associated with the beauty rating. The best model was selected based on the Bayesian Information Criterion (BIC) calculated on the overall fit as well as average BIC after 10-fold cross-validation. (See Supplementary Material for detailed model comparisons in Tables S23–26, and results of all tested models in Tables S27–29.) The results of CLMM models that treated all ratings as ordinal variables yielded the same results as the linear mixed effects models reported below (see Supplementary Tables S30–31).

**Fig. 1.**
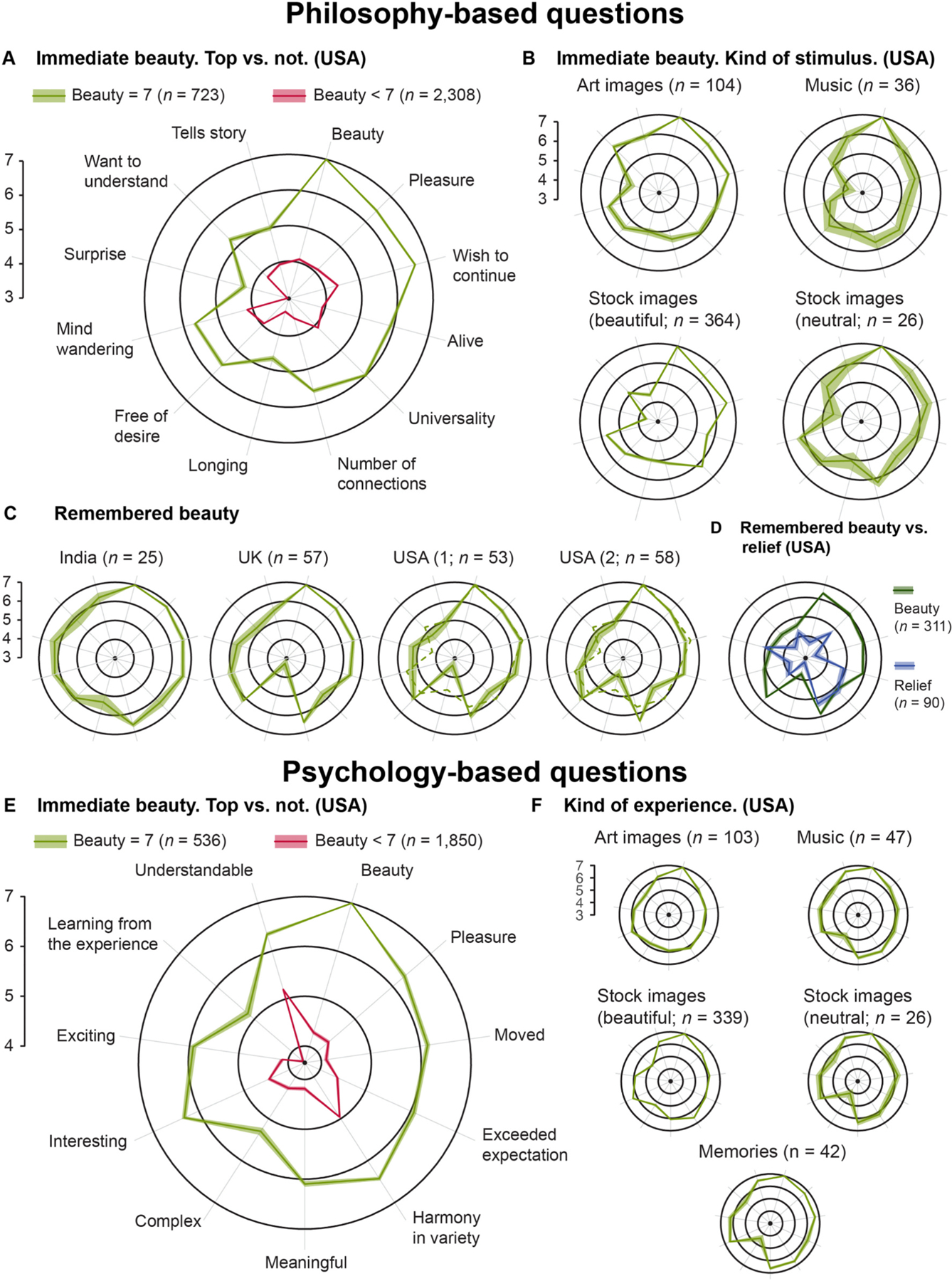
Average ratings for beauty experiences for all experiments. A) In USA, polar plot of average ratings on 12 philosophy-derived dimensions (12D) across all top-rated (green) vs. less-than-top-rated (red) *immediate*-beauty trials (i.e. they consider their immediate experience). B) In USA, average 12D ratings for top-rated immediate-beauty trials, separately for each stimulus type. C) Average 12D ratings on top-rated *remembered*-beauty trials (i.e. they consider their own remembered experience), separately for each independent population sample: India, UK, and two in USA. For reference, the dashed green lines represent the averages for the top-beauty-rated trials, copied from panel A. D) In USA, average ratings for all remembered-*beauty* (dark green) vs. remembered-*relief* (blue) trials. Not pictured: ratings on the two dimensions perfection and peacefulness that were not included in all studies; both peacefulness and perfection ratings were higher for beauty compared to relief ratings. E) In USA, polar plot of average ratings on 11 psychology-derived dimensions (11D) across all top-rated (green) vs. less-than-top-rated (red) *immediate*-beauty trials. F) Average 11D ratings on top-rated immediate and remembered (bottom-most) trials. Shaded areas in all polar plots indicate ±*SEM* (not visible due to small *SEM* in some panels).

Beauty increased primarily with: perceived universality (0.23 points per point), pleasure (0.21 points per point), and a reported wish to continue the experience (0.20 points per point). Beauty was not related to surprise or the degree to which the participant felt that the stimulus told a story. Of note, the type of stimulus (music, image, or specific image kind) did not modulate these effects. Overall, this model explains 72% of the variance. Table 1 presents the statistics of the fit. For illustration, Fig. 1A contrasts the rating profile for stimuli rated highest (7) in beauty vs. that for those rated lower (*<* 7).

### 3.2. Variables correlated with remembered beauty

One might wonder whether a beauty experience produced in an online-test session by a hit (i.e. high average survey rating) image or popular music is likely to be strong enough to be comparable with the intense beauty experiences that philosophers wrote about. We imagine that a philosopher describing the feeling of beauty would naturally emphasize his recollection of his own most intense beauty experience. In that spirit, we asked participants in our second set of studies (Experiment 2a *n* = 92; replication Experiment 2b *n* = 89; both in USA) to describe and rate a remembered intense beauty experience from their own lives. Since we intentionally collected data only for memories of intense beauty, we did not use linear models to explain beauty ratings as in Experiment 1, which would be underpowered due to the small variability in beauty ratings. Instead, we compared the ratings based on top-rated remembered- vs. immediate-beauty trials (*n* = 111 vs. 530). We here report comparisons among the five dimensions correlated with beauty in Experiment 1. Comparisons between ratings on the dimensions not correlated with intense beauty can be found in the Supplementary Material.

According to Kolmogorov-Smirnov tests, ratings of pleasure, wishing to continue the experience, and mind wandering were not differently distributed for immediate- vs. remembered-beauty trials, both *p* ≥ 0.448. Yet, participants rated remembered-beauty higher in terms of feeling alive, feeling free of desire, and the number of felt connections, all 0.41 ≤ *d* ≤ 0.63. In contrast, they rated remembered beautiful experiences as less universally beautiful, wanted to understand them more to a lesser extent, and reported less longing compared to immediate beauty experiences, all 0.32 ≤ *d* ≤ 0.68. Nonetheless, ratings on seven of the nine dimensions correlated with beauty ratings in Experiments 1a and 1b were also high for remembered beauty, all means ≥5.43 on a 1–7 scale. The two exceptions were ratings of wanting to understand the experience more and feeling alive, both means ≤0.76, rendering these dimensions less likely to be essential constituents of beauty experiences. This is unsurprising given the weak association with beauty revealed by the general linear model.

Fig. 1A vs. C illustrate the similarity of average ratings in Experiments 1 vs. 2: immediate vs. remembered beauty. (To facilitate this comparison, the top-beauty data from Fig. 1A appear as dashed lines in the USA panels of Fig. 1C.)

### 3.3. Correlation between survey data and philosophers’ claims

Table 2 lists the complete results of our comparison between philosophers’ claims and the data obtained across all studies. The strength of each dimension’s relation to beauty was scored based on the estimates of the linear mixed-effects model shown in Table 1. Philosophers’ positions were scored by the authors. Of the seven philosophers whose statements we scored on these potentially beauty-related dimensions, our survey aligns best with Kant, with a 74% correlation.

### 3.4. Conservation across cultures

In our third set of studies, we asked how well our previous findings generalize across countries and cultures. We therefore compared the beauty memory ratings from our initial US samples to those from the UK (*n* = 78) and India (*n* = 52). (Our questionnaire is in English, so we confined ourselves to English-speaking countries.) There were differences in the average rating pattern between countries, *F*(2,304) = 3.50, *p <* 0.001. Because average beauty ratings differed, too, we restricted the comparison between countries to top-rated remembered-beauty trials (*n* = 25 for India; *n* = 56 for UK; *n* = 111 for USA). Of the seven dimensions consistently associated with high beauty ratings, only two differed across countries. The impression of universality was higher in India, *M* = 6.36, than in the USA, *M* = 5.43, *p* = 0.018, *d* = 0.62, and reported mind wandering was lower in the USA, *M* = 5.37, than the UK, *M* = 6.05, and India, *M* = 6.32, both *p* ≤ 0.024, both *d* ≥ 0. 41. Notably, longing ratings were much higher in India, *M* = 5.44, than any other country, both *M* ≤ 3.67, both *p* ≤ 0.001, suggesting a greater import of this dimension in India than other English-speaking countries. In sum, the general pattern of ratings correlated with beauty was nearly identical across cultures.

### 3.5. Uniqueness of the beauty-rating profile: a comparison to relief

So far, we have described the characteristics of a beauty experience, but we have not addressed which of these characteristics might distinguish beauty from other positive experiences. To do so, we asked an independent sample of US Americans (*n* = 90) to recall and then describe and rate a personal experience of intense relief. We here compare beauty to relief because it is a strongly positive emotion, the memory of which is not usually also deemed beautiful (in contrast to, e. g., joy, see Supplementary Material). The ratings for remembered relief are unlike those for remembered beauty (see Fig. 1D), *F*(1,395) = 0.53, *p <* 0.001. Follow-up *t*-tests indicate that the two concepts do not differ in perceived universality, *p* = 0.381, or surprise, *p* = 0.471. However, remembered relief received lower ratings on all 12 remaining dimensions, all *p* ≤ 0.001, all *d* ≥ 0.39. Thus, among the dimensions correlated with intense beauty, all but one — universality — are *uniquely* correlated with beauty, not just any positive memory.

### 3.6. Comparison between responses and psychological theories

Our first series of studies focused on questions based on the beauty theories of seven philosophers’ who all make statements about a wide range of characteristics. Next, we wanted to assess how well modern psychological theories of beauty predict people’s beauty experiences. So we ran modified versions of Experiments 1a, 1b, and 2b with 11 questions newly gleaned from psychological theories about beauty (see Table 3 for a complete list).

As before, we assessed which of the 11 tested dimensions were associated with beauty ratings using mixed-effects linear models for data of those participants who rated images and/or music (total *N* = 186). As in Experiment 1, we tested several models. Again, we created two sets of models: In one set, complexity and excitement were added as linear terms, like the other dimensions. In the other set – based on Berlyne’s claims (1971) – they were added as squared terms. According to the average BIC based on 10-fold cross-validation, a model that includes an interaction with stimulus modality explains beauty ratings best, explaining 70% of the variance (see Supplementary Tables S32–33 for detailed model comparisons). For all stimuli, beauty increases with pleasure (0.28 points per pleasure point for images, 0.07 for music), feeling moved (0.18 beauty points per point), feeling that the experience exceeded expectation (0.11 points), perceived harmony among variety (0.11 beauty-points per point), and meaningfulness (0.08 points for images, 0.21 points for music). In addition, beauty ratings were inversely correlated with squared complexity ratings. Of note, interest was correlated with beauty ratings for images, but not for music. The detailed parameters of the model are listed in Table 3. The results of CLM models where all ratings were treated as ordinal variables yielded the same results reported above (see Supplementary Tables S34–35).

Next, in an independent sample (*N* = 72), we compared ratings on the dimensions of immediate experience to those of the remembered beauty experience. Ratings of pleasure, feeling that the experience exceeded expectation, and harmony in variety did not differ between top-rated (=7) stimulus- and memory-related beauty experiences, all *p ≥* 0.265. Memories were, however, rated slightly more moving, meaningful, and relatively less complex, all *p* ≤ 0.040. Of note, all these differences were small, all |*d*| ≤ 0.45, and all means were in the range expected based on the linear model (see Table 3). Thus, as with the beauty-related dimensions from philosophical theories, those derived from psychological theories did not differ much between modalities.

### 3.7. Text analyses

To achieve a characterization of beauty experiences independent of our rating scales, we analyzed the beauty memory descriptions that our participants wrote down before rating them (available with all data at https://github.com/aenneb/characterizing_beauty). Table 4 lists the top ten most frequently used words (excluding stop-words) for each experiment (extracted using NLTK; Loper & Bird, 2002). Going beyond the count of word frequencies, we used the *empath client* (Bates et al., 2015) to analyze which lexical categories are most represented in our beauty descriptions compared to a standard text corpus. We find that the top ten lexical categories in the beauty memory descriptions were, in order: beauty, attractive, feminine, weather, children, love, beach, vacation, positive emotion, and party. These stand in stark contrast to most of the top themes emerging in relief memories, i.e., negative emotion, contentment, joy, traveling, party, family, driving, home, listen, pain. Thus, the remembered experiences were typically active and social, like a family holiday, unlike the solitary appraisal of art emphasized in writings on aesthetics.

The Supplementary Material contains detailed text analyses for each experiment. Writings in aesthetics emphasize solitary appraisal of art, but the beautiful experience recalled by our participants was more typically active and social, like a family holiday.

### 3.8. People’s explicit beliefs about beauty

**Fig. 2.**
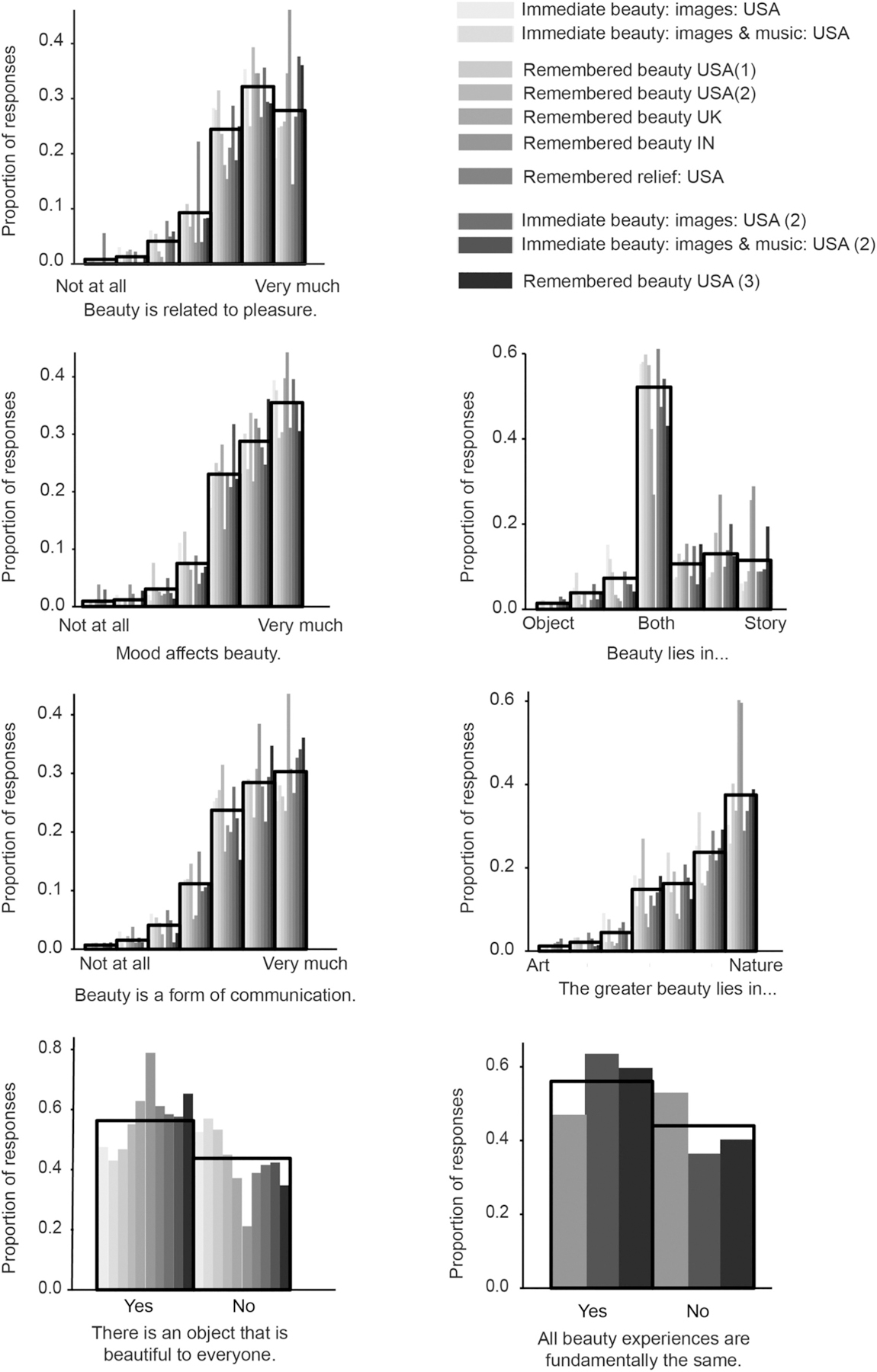
Histograms of ratings in response to the seven questions on participants’ general beliefs about beauty posed at the end of each experiment. Each solid bar indicates the proportion per experiment, differentiated by shades of gray. Immediate beauty USA (2) and remembered beauty USA (3) refers to the people who rated psychology-based questions. Open bars indicate the overall distribution of ratings across all experiments.

Participants in all experiments answered the same six questions about their general beliefs about beauty at the end of the experiment (total *n* = 851). As illustrated in Fig. 2, participants in all countries did endorse the statement that pleasure and beauty are closely related, that sharing beauty is a form of communication, and that mood influences beauty, *Md* = 6 each on a 1 (not at all) to 7 (very much) scale. They also perceived beauty to lie in nature more than in art, *Md* = 6 on a 1 (art) to 7 (nature) scale, and both within the object itself as well as in the story it tells, *Md* = 4. The only question that divided people was whether a universally beautiful object exists: About half of our participants (56.29%) agreed, the remainder did not. We asked those participants to tell us of such an object. Their answers fell into a few categories. The great majority (78% of 479) named an element of nature, mostly flowers (22%), or the sky, sun, and related phenomena (22%). Of the much rarer non-nature-related answers, the most common ones referred to valuable objects (6%), mostly diamonds, or artworks (broadly construed: 6%). Participants who answered questions based on psychological theories (*N* = 258) were also asked whether all beauty experiences are fundamentally the same, since this statement is the central claim emerging from a series of fMRI studies (Ishizu & Zeki, 2011). About half of these participants agreed with the statement (56%). This answer was somewhat related to people’s belief in a universally beautiful object. 64% of participants who said there is a universally beautiful object also said that all beauty experiences are the same, whereas only 44% of those who did not believe in a universally beautiful object said that all beauty experiences are the same.

### 3.9. Individual differences

The main goal of this article is to provide an overview of the characteristics of beauty experience that are conserved across individuals. However, several studies have shown that less than a third of the variance in beauty ratings for anything but faces can be explained by shared taste across observers (Brielmann & Pelli, 2019; Vessel et al., 2018). Therefore, we also want to provide a quick overview of individual differences that modify our population-wide statements. To do so, we looked at whether the relationship between ratings of beauty and the other assessed dimensions differed between participants who rated more than one experience. To that end we used cluster-analyses to determine which participants in Experiments 1 and 5 showed consistent patterns of correlations between beauty and other ratings (see Supplementary Material for detailed analysis descriptions and results).

For both experiments, participants fell into two clusters. In both cases, the first cluster was the larger one (*N* = 112 and *N* = 123, respectively) and showed a pattern like the linear mixed-effects model (see Tables 1, 3). The smaller clusters in both experiments (*N* = 64 and *N* = 51, respectively) consisted of participants whose beauty ratings were only weakly correlated with ratings on the other dimensions. Demographic variables and answers to most general questions were unrelated to cluster assignment. We only found differences between the two clusters in data from Experiment 5, where Participants in cluster 2 were on average 8 years younger, more likely to state that a universally beautiful object exists (72% vs. 46% yes responses), and attributed an image’s beauty slightly more to the “story it tells” than the image itself. Otherwise, we found no differences between clusters, all *p* ≥ 0.159.

In sum, the cluster analyses suggest that there is a smaller subpopulation for which the relationship between beauty and other experiences is less pronounced that for the majority. Whether this subpopulation differs in terms of its demographics and further aspects of aesthetic experiences remains to be determined given ambiguous results across experiments in our studies and the exploratory nature of the analyses.

## 4. Discussion

### 4.1. Survey vs. philosophers: model explains 72% of variance

We identified 11 feeling-of-beauty dimensions in the writings of seven philosophers. Our philosophical mixed-effects model uses those dimensions to account for 72% of the variance in beauty ratings. Compared to our survey results, of the seven philosophers considered here, Kant’s claims are the most correlated (*r* = 0.74), and have the most matches to the data: 10 out of 11 assessed dimensions (see Table 2). Kant’s theory also states our participants’ belief that beauty is found in nature rather than art and that both the object as well as its story contributes to beauty. Yet, contrary to Kant’s theory, higher surprise is not associated with more intense beauty. However, as we will show below, there is a kind of surprise, i.e., the surprise of something exceeding one’s expectation, that is indeed linked to beauty.

### 4.2. Survey vs. psychologists: model explains 70% of variance

We identified 10 feeling-of-beauty dimensions in the 25 papers and books (by 54 authors) on psychological theories of beauty that we cite here and in the supplement. Our psychological mixed-effects model uses 7 of those dimensions to account for 70% of the variance in beauty ratings. The ratings for images, music, and memories reveal a link to beauty for seven out of the eleven here-considered characteristics that psychologists claim are linked to beauty. In all of our surveys, intense beauty was associated with intense pleasure, as claimed by Fechner (1876). A strong link between beauty and being moved was also evident, as reported by Vessel et al. (2013). The notion that a positive prediction error contributes to beauty (Salimpoor et al., 2015) was also confirmed. Harmony in variety, the central beauty criterion in Diessner et al.’s (2018) theory was associated with beauty, too, and so was meaningfulness (see Leder & Nadal, 2014). We found mixed results regarding Berlyne’s (1971) claims. While our results indeed suggest an inverted-u-shaped relation between subjective complexity and beauty, where medium complexity levels are associated with most intense beauty, we did not find such a relationship between beauty and arousal (assessed as excitement). Our music results reject Kivy’s (1990) claim that musical beauty is linked to interest.

Information-seeking — i.e. learning, interest, and wanting to understand the experience— was not systematically linked to beauty in our studies, contrary to previous claims (Biederman & Vessel, 2006). Our results reject Reber et al.’s (2004) claim that ease of processing—i.e., felt understanding—of the experience is tied to its beauty, at least not in a way that is consciously accessible to people. The other cognitive dimension we included, learning, was also not associated with beauty, contrary to claims by Armstrong and Detweiler-Bedell’s (2008). Taken together, these negative findings indicate that information-seeking is not important for experiencing beauty, at least not in their awareness.

One might suppose that depiction of an ugly object could only be beautiful by offering information, but we do not believe that the lack of correlation between beauty and self-reported information seeking prevents beautiful depiction of an ugly object. For instance, it seems possible that the mature Rembrandt, seen in person, might have been thought ugly. But his way of painting himself – the brush strokes, color-selection, and so on – may evoke beauty among those admiring his self-portrait.

It is also worth noting that our findings regarding people’s explicit, self-reported feelings of learning progress do not exclude the possibility that beauty or pleasure serves as a signal of unconscious learning progress, e.g., in the sense of increasing long-term processing efficiency (Brielmann & Dayan, 2021).

### 4.3. Implications for the science of beauty

Our results, summarized in Table 5, provide an empirical characterization of the beauty experience. They will inform current theories of aesthetic appreciation (e.g., Leder & Nadal, 2014; Pelowski et al., 2017) and provide a first broad test of philosophy- and psychology-based theories of beauty. Our findings complement previous efforts that contrasted people’s theoretical conceptions of beauty to other aesthetic evaluations (Menninghaus et al., 2019; see Supplementary Material for a quantitative comparison between their and our data).

Our findings are in line with the notion that beauty is a positive emotion, i.e., strongly correlated with pleasure (Armstrong & Detweiler-Bedell, 2008; Fechner, 1876) and being moved (Vessel et al., 2013). We did not find a correlation between intense beauty and information-seeking here, i.e., wanting to understand the experience, learning, or interest, despite the prevalence of these notions in several psychological theories (Armstrong & Detweiler-Bedell, 2008; Kivy, 1990; Reber et al., 2004). We did, however, find that several other features mentioned in contemporary literature are indeed correlated with beauty, such as the feeling that the experience exceeded expectation (Salimpoor et al., 2015), harmoniously combined various elements (Diessner et al., 2018), and meaningfulness (Leder & Nadal, 2014).

Our current study documents the feelings that are correlated with beauty. This list of beauty-associated characteristics offers a basis for developing a predictive model. The development of a model that can predict the beauty intensity of a given experience with as few predictors as possible would be a big step in explaining beauty.

### 4.4. Beauty and art

The philosopher and art critic Arthur Danto (2002) noted that until World War I and the dada movement, it was generally accepted that beauty was central to the definition of art. Danto notes that dada and the subsequent postmodern movements “disconnect[ed] beauty from art”. Art today is much more general than just beauty. Danto says that beauty is merely one of many attributes that art can have and that the only necessary one is meaning. However, Danto’s strict dichotomy between beauty and meaning is undermined by finding that beauty is associated with meaningfulness in our participants’ reports.

Our participants’ beauty memories and beliefs assign hardly any role to art. A typical memory of an intense beauty experience was a family vacation on the beach or the mountains, rather than a museum visit. Participants agreed that beauty lies in nature, not art (see Fig. 2), and most of those who believed in a universally beautiful object thought it was part of nature, like flowers or sky. Only 6% of the intense beauty recollections (31/479) mentioned any kind of art.

Beauty is central to popular notions of aesthetics (Augustin et al., 2012), but, in academia, aesthetics and empirical aesthetics (e.g. Leder & Nadal, 2014; Pelowski et al., 2017) focus on art appreciation. Our results challenge the relevance of art, and thus art appreciation, aesthetics, and empirical aesthetics, to how most people experience beauty.

Here, we set out to characterize people’s notions and experiences of beauty. In our survey sample of ordinary people—neither artists nor academics—we found that moments of intense beauty are associated with nature and social interactions rather than art. We wonder how members of the art world, e.g. art school students, might respond to a feeling-of-beauty survey like ours. Art matters in their daily lives, but the modern movement assigns little role to beauty in modern art (Danto, 2002), so, even in the art world, descriptions of intense beauty experiences might only rarely mention art. Similarly, since we got so few mentions of art in the intense-beauty recollections, we also wonder if the beauty evoked by art might be different, possibly requiring more learning and understanding (Armstrong & Detweiler-Bedell, 2008). We leave that to future work, but our results already suggest that understanding everyday beauty experiences may require a theory of beauty that deals with social and nature-related experiences.

## 5. Conclusion

Surveying what 851 ordinary people feel in top-rated beauty experiences, either immediate or recalled, reveals that the experience of beauty is characterized by: intense pleasure, an impression of universality, wanting to continue the experience, exceeded expectation, perceived harmony in variety, and meaningfulness. This holds true for images, music, and memories across three culturally-diverse English-speaking nations. The remembered experiences were typically active and social, like a family holiday, and only rarely mentioned art, unlike the solitary appraisal of art emphasized in aesthetics. Among seven renowned philosophers of aesthetics, Kant’s definition best matches the results of our feeling-of-beauty survey. Our results are in line with several psychological theories of beauty but not with those that emphasize information seeking.

## Supplementary Material

Supp.Materials

## Figures and Tables

**Table 1:** The mixed-effects model that best explains rated beauty of images and music.

Random effects							
		Variance				*SD*	
Participant		0.07				0.26	
Stimulus		0.13				0.37	
Fixed effects							
	Estimate		*SE*	*df*	*t*		*p*

Intercept	**0.34**		**0.12**	**65**	**2.93**		**0.005**
1. Universality	**0.23**		**0.02**	**2212**	**12.23**		**<0.001**
2. Pleasure	**0.21**		**0.02**	**2584**	**11.04**		**<0.001**
3. Wish to continue	**0.20**		**0.02**	**2622**	**11.27**		**<0.001**
4. Feeling alive	**0.08**		**0.02**	**2645**	**4.30**		**<0.001**
5. Feeling free of desire	**0.07**		**0.02**	**2444**	**4.25**		**<0.001**
6. Number of felt connections	**0.06**		**0.02**	**2393**	**3.81**		**<0.001**
7. Mind wandering	**0.05**		**0.02**	**2440**	**3.42**		**0.001**
8. Wanting to understand more^a^	**0.04**		**0.02**	**2567**	**2.76**		**0.006**
9. Longing^a^	**0.04**		**0.02**	**2134**	**2.38**		**0.017**
10. Telling a story	‒0.01		0.01	2393	‒0.73		0.466
11. Surprise	‒0.02		0.01	1882	‒1.13		0.260

Notes. Significant fixed effects are highlighted in bold. The model explains 72% of the variance in beauty ratings (on a scale of 1 to 7) with an RMSE of 0.96.

a The marked dimensions did not receive consistently high ratings for remembered beauty, which shows that they are not essential for feeling beauty.

**Table 2:** Survey vs. philosophers’ claims.

Dimension	Survey	Plato	Aristotle	Hume	Kant	Hegel	Santayana	Danto
	2019	425 BCE	384 BCE	1711	1724	1770	1863	1924
1. Universality	2	2	1	0	2	1	0	2
2. Pleasure	2	2	2	2	2	0	2	2
3. Wish to continue	2	1	1	2	2	2	0	0
4. Feeling alive	1	2	2	2	2	2	0	1
5. Feeling free of desire	1	0	0	0	2	0	1	2
6. Number of felt connections	1	0	1	1	1	2	1	2
7. Mind-wandering	1	2	1	1	1	1	1	1
8. Wanting to understand more	1	1	1	1	2	2	0	2
9. Longing	1	2	0	0	1	0	1	0
10. Telling a story	0	0	2	1	0	2	0	1
11. Surprise	0	1	2	1	1	2	‒1	0
Correlation *r*		0.46	‒0.22	0.18	0.74	‒0.36	0.44	0.30

Notes. Black/red integers signify strength of observed (in survey) or posited (by philosophers) association with beauty: 2 strongly positive, 1 positive; 0 none or not mentioned; ‒1 negative. For the survey, positive relations according to Table 1 indicating an increase of at least 0.2 points in beauty per point on the dimension in question were coded as 2, significant positive increases as 1, and non-significant relations as 0. Each correlation coefficient *r* in the last row is the Pearson correlation of two columns, survey vs. philosopher. The second row denotes the year of our survey and the birth year of each philosopher.

**Table 3:** The mixed-effects model that best explains rated beauty of images and music based on questions from psychological theories.

Random effects							
			Variance			*SD*	
Participant			0.06			0.25	
Stimulus			0.05			0.21	
Fixed effects							
	Estimate	*SE*		*df*	*t*		*p*

(Intercept)	**0.55**	**0.13**		**287**	**4.09**		**<0.001**
Music	**‒0.49**	**0.27**		**312**	**‒1.86**		**0.063**
Pleasure	**0.28**	**0.02**		**2221**	**12.00**		**<0.001**
Moved	**0.18**	**0.02**		**2229**	**8.09**		**<0.001**
Exceeded expectation	**0.11**	**0.02**		**2148**	**5.05**		**<0.001**
Harmony in variety	**0.11**	**0.02**		**2219**	**5.44**		**<0.001**
Meaningful	**0.08**	**0.02**		**2227**	**3.34**		**0.001**
Complex (squared)	**‒0.02**	**0.01**		**2192**	**‒2.58**		**0.010**
Interesting	**0.17**	**0.02**		**2225**	**7.61**		**<0.001**
Exciting (squared)	‒0.01	0.01		2215	‒0.81		0.418
Learning from the experience	‒0.02	0.02		1482	‒1.13		0.257
Understandable	0.03	0.02		2144	1.53		0.127
Music × pleasure	**‒0.21**	**0.06**		**2204**	**‒3.64**		**<0.001**
Music × moved	‒0.03	0.06		2222	‒0.57		0.568
Music × exceeded expectation	‒0.02	0.05		2212	‒0.47		0.638
Music × harmony in variety	‒0.07	0.05		2236	‒1.37		0.170
Music × meaningful	**0.14**	**0.05**		**2228**	**2.52**		**0.012**
Music × complex (squared)	0.01	0.02		2226	0.29		0.773
Music × interesting	**‒0.15**	**0.06**		**2227**	**‒2.76**		**0.006**
Music × exciting (squared)	0.00	0.02		2223	0.07		0.941
Music × learning from the experience	**0.29**	**0.04**		**2217**	**6.53**		**<0.001**
Music × understandable	0.08	0.05		2230	1.59		0.113

Notes. Significant fixed effects are highlighted in bold. The model explained 70% of the variance in beauty ratings (on scale of 1 to 7) with an RMSE of 0.94.

**Table 4:** Top-ten most frequently appearing words across beauty memory descriptions per experiment.

Frequency rank	Exp 2a (USA)	Exp 2b (USA)	Exp 3a (UK)	Exp 3b (India)	Exp6 (USA)	All
1	Beautiful	Beautiful	Day	Beautiful	Beautiful	Beautiful
2	Experience	Beauty	Beautiful	Felt	Time	Day
3	Could	Felt	Time	Feel	Day	Time
4	Like	Time	Could	Day	Beauty	Beauty
5	Beauty	Day	Like	Beauty	Remember	Like
6	See	Like	See	Time	One	Felt
7	One	Went	Felt	Like	See	One
8	Time	Nature	Smell	Experience	Went	See
9	Went	One	Beauty	Life	Mountains	Went
10	Remember	First	Sun	Place	Feel	Could

Notes. Counts for each word per experiment are provided in the Supplementary Material.

**Table 5:** The significant fixed effects in the mixed-effects models of beauty drawn from philosophy (Table 1) and psychology (Table 3).

From philosophy	From psychology	Combined
Accounts for 72% of beauty variance.	Accounts for 70% of beauty variance.	Strongest fixed effects across studies
Pleasure	Pleasure	Pleasure
Universality	Music	Universality
Wish to continue	Moved	Wish to continue
Feeling alive	Exceeded expectation	Exceeded expectation
Feeling free of desire	Harmony in variety	Harmony in variety
Number of felt connections	Meaningful	Meaningful
Mind wandering	Complex (squared)	
Wanting to understand more	Interesting	
Longing	Music × pleasure	
	Music × meaningful	
	Music × interesting	
	Music × learning from the experience	

## References

[R1] Armstrong T, & Detweiler-Bedell B (2008). Beauty as an emotion: The exhilarating prospect of mastering a challenging world. Review of General Psychology, 12(4), 305–329.

[R2] Augustin MD, Wagemans J, & Carbon C-C (2012). All is beautiful? Generality vs. specificity of word usage in visual aesthetics. Acta Psychologica, 139(1), 187–201. 22123506 10.1016/j.actpsy.2011.10.004

[R3] Bartoń K (2019). MuMIn: Multi-model inference. R package version 1.43.6. https://CRAN.R-project.org/package=MuMIn.

[R4] Bates D, Maechler M, Bolker B, & Walker S (2015). Fitting linear mixed-effects models using lme4. Journal of Statistical Software, 67(1), 1–48.

[R5] Belfi AM, Vessel EA, Brielmann A, Isik AI, Chatterjee A, Leder H, Pelli DG, & Starr GG (2019). Dynamics of aesthetic experience are reflected in the default-mode network. Neuroimage, 188, 584–597. 30543845 10.1016/j.neuroimage.2018.12.017 PMC8493917

[R6] Berlyne DE (1971). Aesthetics and psychobiology. New York, NY: Appleton-Century-Crofts.

[R7] Berlyne DE (1974). Studies in the new experimental aesthetics: Steps toward an objective psychology of aesthetic appreciation. Washington, DC: Hemisphere.

[R8] Biederman I, & Vessel EA (2006). Perceptual pleasure and the brain: A novel theory explains why the brain craves information and seeks it through the senses. American Scientist, 94(3), 247–253.

[R9] Brielmann A, & Dayan P (2021). Introducing a computational model of aesthetic value. 10.1037/rev0000337 35786988

[R10] Brielmann AA (2020, 6 11). Preschool of aesthetics translated by Brielmann Aenne A. https://aenneb.github.io/files/Preshool%20of%20aesthetics%20by%20Aenne%20A%20Brielmann.pdf.

[R11] Brielmann AA, & Pelli D (2019). Intense beauty requires intense pleasure. Frontiers in Psychology, 10, 2420. 31749737 10.3389/fpsyg.2019.02420 PMC6848232

[R12] Brielmann AA, & Pelli DG (2018). Aesthetics. Current Biology, 28(16), R859–R863. 30130500 10.1016/j.cub.2018.06.004

[R13] Danto AC (2002). The abuse of beauty. Daedalus, 131(4), 35–56. On Beauty (Fall, 2002) http://www.jstor.org/stable/20027805.

[R14] Denham AE (Ed.). (2012). Plato on art and beauty. New York, NY: Palgrave MacMillan.

[R15] Diessner R, Pohling R, Stacy S, & Güsewell A (2018). Trait appreciation of beauty: A story of love, transcendence, and inquiry. Review of General Psychology, 22(4), 377–397.

[R16] Fast E, Chen B, & Bernstein MS (2016, 5). Empath: Understanding topic signals in large-scale text. In Proceedings of the 2016 CHI conference on human factors in computing systems (pp. 4647–4657). ACM.

[R17] Fechner GT (1876). Vorschule Der Aesthetik. Leipzig: Breitkopf and Haertel.

[R18] Halliwell S (1986). Aristotle’s poetics. Chapel Hill, NC: University of North Carolina Press.

[R19] Hume. (1739). A treatise of human nature. Retrieved online from https://davidhume.org/texts/t/2/1/8.

[R20] Ishizu T, & Zeki S (2011). Toward a brain-based theory of beauty. PLoS One, 6(7). 10.1371/journal.pone.0021852 PMC3130765 21755004

[R21] Kivy P (1990). Music alone: Philosophical reflections on the purely musical experience. Cornell University Press.

[R22] Koelsch S, Vuust P, & Friston K (2019). Predictive processes and the peculiar case of music. Trends in Cognitive Sciences, 23(1), 63–77. 30471869 10.1016/j.tics.2018.10.006

[R23] Kurdi B, Lozano S, & Banaji MR (2017). Introducing the open affective standardized image set (OASIS). Behavior Research Methods, 49(2), 457–470. 26907748 10.3758/s13428-016-0715-3

[R24] Kuznetsova A, Brockhoff PB, & Christensen RHB (2017). lmerTest package: Tests in linear mixed effects models. Journal of Statistical Software, 82(13), 1–26.

[R25] Leder H, & Nadal M (2014). Ten years of a model of aesthetic appreciation and aesthetic judgments: The aesthetic episode–developments and challenges in empirical aesthetics. British Journal of Psychology, 105(4), 443–464. 25280118 10.1111/bjop.12084

[R26] Loper E, & Bird S (2002). NLTK: The natural language toolkit. arXiv preprint cs/0205028.

[R27] Marin MM, Lampatz A, Wandl M, & Leder H (2016). Berlyne revisited: Evidence for the multifaceted nature of hedonic tone in the appreciation of paintings and music. Frontiers in Human Neuroscience, 10, 536. 27867350 10.3389/fnhum.2016.00536 PMC5095118

[R28] Menninghaus W, Wagner V, Kegel V, Knoop CA, & Schlotz W (2019). Beauty, elegance, grace, and sexiness compared. PLoS One, 14(6), Article e0218728. 10.1371/journal.pone.0218728 PMC6588248 31226137

[R29] Nuzzo A (2005). Kant and the Unity of reason. West Lafayette, IN: Purdue University Press.

[R30] Nuzzo A (2006). Hegel’s “aesthetics” as theory of absolute spirit. Internationales Jahrbuch des Deutschen Idealismus/International Yearbook of German Idealism, 4, 291–310.

[R31] Palmer SE, Schloss KB, & Sammartino J (2013). Visual aesthetics and human preference. Annual Review of Psychology, 64, 77–107. 10.1146/annurev-psych-120710-100504 23020642

[R32] Pelowski M, Markey PS, Forster M, Gerger G, & Leder H (2017). Move me, astonish me… delight my eyes and brain: The Vienna integrated model of top-down and bottom-up processes in art perception (VIMAP) and corresponding affective, evaluative, and neurophysiological correlates. Physics of Life Reviews, 21, 80–125. 28347673 10.1016/j.plrev.2017.02.003

[R33] Reber R, Schwarz N, & Winkielman P (2004). Processing fluency and aesthetic pleasure: Is beauty in the perceiver’s processing experience? Personality and Social Psychology Review, 8(4), 364–382. 15582859 10.1207/s15327957pspr0804_3

[R34] Rhodes G (2006). The evolutionary psychology of facial beauty. Annual Review of Psychology, 57, 199–226. 10.1146/annurev.psych.57.102904.190208 16318594

[R35] Salimpoor VN, Zald DH, Zatorre RJ, Dagher A, & McIntosh AR (2015). Predictions and the brain: How musical sounds become rewarding. Trends in Cognitive Sciences, 19(2), 86–91. 25534332 10.1016/j.tics.2014.12.001

[R36] Santayana G (1955). The sense of beauty: Being the outline of aesthetic theory (Vol. 238). Courier Corporation.

[R37] Santayana G (1896). The sense of beauty. In Being the outlines of aesthetic theory. New York: Charles Scribner’s Sons.

[R38] Taylor J (2008). Hume. In: Hume on beauty and virtue. Radcliff E. (Ed.). Oxford: Blackwell.

[R39] Vessel EA, Maurer N, Denker AH, & Starr GG (2018). Stronger shared taste for natural aesthetic domains than for artifacts of human culture. Cognition, 179, 121–131. 29936343 10.1016/j.cognition.2018.06.009

[R40] Vessel EA, Starr GG, & Rubin N (2013). Art reaches within: Aesthetic experience, the self and the default mode network. Frontiers in Neuroscience, 7, 258. 24415994 10.3389/fnins.2013.00258 PMC3874727

[R41] Wittgenstein L (1938). Lectures & conversations on aesthetics. In Psychology and religious belief. Berkeley and Los Angeles: University of California Press.

