# Supplementary material for: Beauty, the feeling
Source: Acta Psychol (Amst). Author manuscript; Available in PMC 2021 Oct 14. (PMC8514293; doi:10.1016/j.actpsy.2021.103365)
Supplement: Supp.Materials [file NIHMS1722925-supplement-Supp_Materials.docx]

**Beauty, the feeling**

**Supplementary Material**

Data availability 3

Generation of questions 3

Verbatim instructions and questions 3

General beliefs about beauty questions. 3

Experiment 1. 4

Experiments 2 -4. 5

Experiments 5 & 6. 7

Experiment 1a: additional analyses 7

Separate linear models for Experiment 1a 7

Comparison to data from Menninghaus et al. (2019) 22

Experiment 1b: separate linear models 22

Combined data from Experiment 1a and 1b: additional analyses 32

Model comparisons 32

Additional linear mixed effects models tested 34

Additional cumulative link mixed models tested 36

Cluster analyses 38

Experiment 2a &2b: Beauty memories (USA) 39

Experiment 2a: text analysis. 39

Experiment 2b: additional demographic information. 40

Experiment 2b: text analysis. 40

Experiment 2b: Perceptual task. 41

Comparison between Experiment 2a & 2b 42

Additional comparisons between Experiment 1 and 2 43

Experiment 3a: Beauty memories in the UK 43

Text analysis. 44

Experiment 3b: Beauty memories in India 45

Perceptual task. 46

Text analysis. 47

Experiment 5 48

Experiment 5a: additional demographic information 48

Model comparisons 48

Additional cumulative link mixed models tested 49

Cluster analyses 51

Experiment 6 52

Additional demographic information 52

Text analysis. 53

Additional experiment: comparing beauty and joy memories 53

Methods 53

Results 53

References 55

# Data availability

All data, including the full descriptions of remembered beauty our participants provided, and main analyses files for this article are accessible on GitHub: <https://github.com/aenneb/characterizing_beauty>.

# Generation of questions

All questions pertaining to philosophers’ theories were based on discussions of all three authors, one of which is a distinguished philosophy scholar with particular expertise on Kant’s and Hegel’s aesthetics (Nuzzo, 2005; 2006). Accordingly, we generated questions based on the following rationale: “What would [Kant / Hegel] ask a person to find out whether she had a genuine beauty experience?”. Special additional consideration was given to the positions of Aristotle (Haliwell, 1989), Plato (Denham, 2012), and Hume (Taylor, 2008).

Questions pertaining to psychologist’s theories were based on the articles published by them. They were initially collected by one of the authors and then discussed with the entire lab. The most relevant reference article for each question is listed along with each verbatim question below.

# Verbatim instructions and questions

## General beliefs about beauty questions.

The following questions were shown to participants in all experiments at the end of their respective different surveys.

1. How closely related are the feelings of beauty and pleasure?
   Not at all (1) to Very closely (7)
2. Which is greater, the beauty of art or nature?
   Art (1) to Nature (7)
3. The beauty of an image is…
   Entirely in the image (1) to Entirely in the story the image tells you (7)
4. Are shared experiences of beauty a form of communication?
   Not at all (1) to Very much (7)
5. How much does mood affect the feeling of beauty?
   Not at all (1) to Very much (7)
6. Can you name an object that everyone finds beautiful?
   Yes or No
7. [If yes for 6.] Please name the object(s) that everyone finds beautiful.
8. [participants in the psychology-theory surveys only, Experiments 5 & 6] The experience of beauty is fundamentally the same, no matter whether it is caused by an image, music, or any other kind of object.
   Yes or No (Ishizu & Zeki, 2011)

## Experiment 1.

The instructions read as follows: “Thank you for participating in our study. In the following, you will answer a series of questions about some images. We will always show you one question and one image at a time. 
You will answer all questions on a scale from "not at all" to "very much". You can use either of these extremes or any point in between to indicate a less extreme opinion.
In answering the question, please refer to what you feel and think as you look at the image.
We are interested in your personal opinion. There are no right or wrong answers.”

Afterwards, participants answered each of the following questions for each image, in random order:

1. How much beauty do you feel from looking at this image right now?
2. How much pleasure do you feel from looking at this image right now?
3. Does this image surprise you?
4. Does this image make you want to look at it?
5. As you look at this image, do you feel content, purely contemplative, free of desire?
6. As you look at this image, how alive, excited, do you feel?
7. As you look at this image, do you want to understand it more?
8. As you look at this image, is your mind wandering freely?
9. In how many ways do you feel connected to this image?
10. Does this image tell you a story?
11. Is this image beautiful to everyone?
12. As you look at this image, do you feel longing, unfulfilled desire?

All ratings were given on a (1) Not at all to (7) Very much scale, except for the questions about the number of connections which was answered on a (1) None to (7) scale.

## Experiments 2 -4.

The instructions read as follows: “Please think back to an experience during which you felt intense [Experiment 2-3: beauty; Experiment 4: relief]. Picture the experience. Remember as many details as you can: what you saw, heard, smelled, and felt.

Let the memory linger for a minute.

When you are ready, continue to the next page (you will be able to do so after one minute has elapsed).”

Afterwards, participants answered each of the following questions in random order:

1. How much beauty did you feel during the experience?
2. How much pleasure did you feel during the experience?
3. Did the experience surprise you?
4. Did the experience make you want to experience it for a longer time?
5. As you had the experience, did you feel content, purely contemplative, free of desire?
6. As you had the experience, how alive, excited, did you feel?
7. As you had the experience, did you want to understand it more?
8. As you had the experience, did your mind wander freely?
9. In how many ways did you feel connected to the experience?
10. Did the experience tell you a story?
11. Is the experience [beautiful / a relief] to everyone?
12. As you had the experience, did you feel longing, unfulfilled desire?
13. Did you feel calm and peaceful during the experience?
14. How perfect was this experience?

All ratings were given on a (1) Not at all / none to (7) Very much / many scale.

## Experiments 5 & 6.

The instructions and procedure were identical to the ones in the previous experiments. The participants in these experiments did answer a new series of questions, based on psychological theories of beauty:

1. How pleasurable was the experience? (Fechner, 1876)
2. How complex was the experience? (Berlyne, 1971)
3. How exciting was the experience? (Berlyne, 1971)
4. How much did you learn from the experience? (Armstrong & Detweiler-Bedell, 2008)
5. How understandable was the experience? (Redies, 2014)
6. How harmoniously did the experience combine its various elements? (Diessner, Pohling, Stacy, & Güsewell, 2018)
7. How meaningful was the experience? (Leder, Belke, Oeberst, & Augustin, 2004)
8. How did the experience compare to what you anticipated?
   Much worse (1) - Much better (7) (Salimpoor, Zald, Zatorre, Dagher, & McIntosh, 2015)
9. How interesting was the experience? (Kivy, 1990)
10. How much did the experience move you? (Vessel, Starr, & Rubin, 2013)

# Experiment 1a: additional analyses

## Separate linear models for Experiment 1a

Of all linear mixed-effects models tested (see **Supplementary Tables S1-13**), one that incorporated random effects of participant and image, as well as the interaction between image category and the eleven non-beauty ratings, explained the highest proportion of rating variance, *R*^2^ = 0.77. The model effects are listed in **Supplementary Table S1**. The model that performed best according to Bayes Factor was one that incorporated the interaction with gender instead of image category, *R*^2^ = 0.76. The gender model effects are listed in **Supplementary Table S2**. Notably, all main effects of the other ratings were the same for both models, with the only exception of longing, reaching significance only in the stimulus category model.

Thus, in general, beauty increased with increasing ratings of pleasure, Wish to continue, feeling alive, a feeling that the image is beautiful to everyone, and feeling free of desire. This is equivalent to the findings reported across Experiments 1a and 1b in the main manuscript.

**Table S1. The linear-mixed effects model for Experiment 1a, including stimulus category as an additional factor.**

| Random effects | | | | | |
| --- | --- | --- | --- | --- | --- |
|  | Variance | *SD* |  |  |  |
| Participant | 0.04 | 0.21 |  |  |  |
| Image | 0.004 | 0.07 |  |  |  |
| Fixed effects | | | | | |
|  | Estimate | *SE* | *df* | *t* | *p* |
| Intercept | 0.22 | 0.15 | 552 | 1.41 | 0.159 |
| **Beautiful stock-image (BS)** | **0.76** | **0.24** | **852** | **3.17** | **0.002** |
| Neutral stock-image (NS) | -0.32 | 0.20 | 254 | -1.64 | 0.101 |
| **Pleasure** | **0.25** | **0.03** | **1743** | **7.28** | **<0.001** |
| **Wish to continue** | **0.19** | **0.03** | **1741** | **5.74** | **<0.001** |
| **Feeling alive** | **0.16** | **0.03** | **1596** | **4.67** | **<0.001** |
| **Universality** | **0.12** | **0.03** | **1366** | **3.63** | **<0.001** |
| **Number of felt connections** | **0.08** | **0.03** | **1703** | **2.61** | **0.009** |
| **Longing** | **0.07** | **0.03** | **1701** | **2.55** | **0.011** |
| **Feeling free of desire** | **0.06** | **0.03** | **1725** | **2.06** | **0.040** |
| Mind-wandering | 0.05 | 0.03 | 1724 | 1.56 | 0.120 |
| Surprise | -0.04 | 0.02 | 1442 | -1.59 | 0.113 |
| Wanting to understand more | 0.04 | 0.03 | 1733 | 1.19 | 0.236 |
| Telling a story | 0.03 | 0.03 | 1318 | 1.11 | 0.268 |
| BS × pleasure | -0.01 | 0.05 | 1721 | -0.25 | 0.805 |
| NS × pleasure | 0.03 | 0.06 | 1731 | 0.49 | 0.623 |
| **BS × Wish to continue** | **0.12** | **0.05** | **1743** | **2.34** | **0.019** |
| NS × Wish to continue | -0.08 | 0.06 | 1714 | -1.46 | 0.144 |
| BS × feeling alive | -0.07 | 0.05 | 1716 | -1.51 | 0.132 |
| NS × feeling alive | -0.01 | 0.06 | 1731 | -0.18 | 0.860 |
| BS × Universality | 0.06 | 0.05 | 1641 | 1.13 | 0.260 |
| NS × Universality | 0.06 | 0.05 | 1628 | 1.15 | 0.249 |
| BS × number of felt connections | -0.06 | 0.04 | 1746 | -1.45 | 0.146 |
| **NS × number of felt connections** | **0.12** | **0.05** | **1738** | **2.28** | **0.022** |
| BS × longing | -0.05 | 0.04 | 1737 | -1.24 | 0.215 |
| NS × longing | -0.06 | 0.05 | 1709 | -1.40 | 0.163 |
| BS × feeling free of desire | -0.05 | 0.04 | 1733 | -1.32 | 0.186 |
| NS × feeling free of desire | 0.01 | 0.05 | 1736 | 0.15 | 0.880 |
| BS × mind-wandering | -0.04 | 0.05 | 1720 | -0.98 | 0.330 |
| NS × mind-wandering | 0.00 | 0.04 | 1729 | -0.05 | 0.960 |
| BS × surprise | -0.02 | 0.03 | 1641 | -0.50 | 0.620 |
| NS × surprise | 0.04 | 0.04 | 1618 | 0.97 | 0.334 |
| BS × wanting to understand more | 0.01 | 0.04 | 1742 | 0.13 | 0.897 |
| NS × wanting to understand more | -0.06 | 0.05 | 1707 | -1.15 | 0.250 |
| BS × telling a story | -0.02 | 0.04 | 1549 | -0.56 | 0.576 |
| NS × telling a story | 0.00 | 0.04 | 303 | 0.02 | 0.984 |

*Notes.* The reference level for image category was the beautiful art image category. Significant fixed effects and interactions are highlighted in bold. The model explained 76.5 % of the variance.

**Table S2. The linear-mixed effects model for Experiment 1a, including gender as additional factor.**

| Random effects | | | | | |
| --- | --- | --- | --- | --- | --- |
|  | Variance | *SD* |  |  |  |
| Participant | 0.04 | 0.19 |  |  |  |
| Image | 0.02 | 0.13 |  |  |  |
| Fixed effects | | | | | |
|  | Estimate | *SE* | *df* | *t* | *p* |
| **Intercept** | **0.37** | **0.11** | **191** | **3.32** | **0.001** |
| Gender | -0.17 | 0.18 | 568 | -0.94 | 0.347 |
| **Pleasure** | **0.21** | **0.03** | **1711** | **7.80** | **<0.001** |
| **Wish to continue** | **0.21** | **0.03** | **1613** | **8.12** | **<0.001** |
| **Feeling alive** | **0.18** | **0.03** | **1716** | **6.63** | **<0.001** |
| **Universality** | **0.18** | **0.03** | **1308** | **6.93** | **<0.001** |
| **Number of felt connections** | **0.09** | **0.02** | **1444** | **4.05** | **<0.001** |
| Longing | 0.04 | 0.02 | 1320 | 1.86 | 0.064 |
| **Feeling free of desire** | **0.06** | **0.02** | **1424** | **2.48** | **0.013** |
| Mind-wandering | 0.02 | 0.02 | 1693 | 0.92 | 0.357 |
| Surprise | -0.02 | 0.02 | 885 | -1.08 | 0.281 |
| Wanting to understand more | 0.00 | 0.02 | 1584 | 0.18 | 0.855 |
| Telling a story | -0.01 | 0.02 | 1031 | -0.53 | 0.596 |
| **Gender × pleasure** | **0.11** | **0.05** | **1702** | **2.22** | **0.027** |
| **Gender × Wish to continue** | **0.09** | **0.05** | **1641** | **2.02** | **0.044** |
| **Gender × Feeling alive** | **-0.13** | **0.05** | **1695** | **-2.85** | **0.004** |
| Gender × Universality | 0.00 | 0.05 | 1410 | 0.01 | 0.992 |
| Gender × Number of felt connections | -0.04 | 0.04 | 1460 | -1.06 | 0.291 |
| Gender × Longing | -0.03 | 0.04 | 1256 | -0.81 | 0.420 |
| Gender × Feeling free of desire | -0.04 | 0.04 | 1374 | -0.99 | 0.325 |
| Gender × Mind-wandering | 0.06 | 0.04 | 1451 | 1.37 | 0.173 |
| Gender × Surprise | -0.03 | 0.03 | 1133 | -0.87 | 0.386 |
| Gender × Wanting to understand more | 0.06 | 0.04 | 1643 | 1.58 | 0.115 |
| Gender × Telling a story | -0.01 | 0.03 | 1602 | -0.24 | 0.810 |

*Notes.* The reference level for gender was male. Significant fixed effects and interactions are highlighted in bold. The model explained 75.6 % of the variance.

**Table S3. The linear-mixed effects model for Experiment 1a, without interactions.**

| Random effects | | | | | |
| --- | --- | --- | --- | --- | --- |
|  | Variance | *SD* |  |  |  |
| Participant | 0.05 | 0.21 |  |  |  |
| Image | 0.02 | 0.14 |  |  |  |
| Fixed effects | | | | | |
|  | Estimate | *SE* | *df* | *t* | *p* |
| **Intercept** | **0.34** | **0.10** | **134.9** | **3.3** | **0.001** |
| **Beautiful stock-image (BS)** | **0.26** | **0.02** | **1757.0** | **11.3** | **<0.001** |
| **Neutral stock-image (NS)** | **-0.03** | **0.02** | **1067.0** | **-2.1** | **0.036** |
| **Pleasure** | **0.24** | **0.02** | **1633.0** | **10.9** | **<0.001** |
| **Wish to continue** | **0.13** | **0.02** | **1758.0** | **5.8** | **<0.001** |
| **Feeling alive** | **0.04** | **0.02** | **1666.0** | **1.9** | **0.063** |
| **Universality** | **0.34** | **0.10** | **134.9** | **3.3** | **0.001** |
| **Number of felt connections** | **0.18** | **0.02** | **1323.0** | **8.1** | **<0.001** |
| **Longing** | **0.26** | **0.02** | **1757.0** | **11.3** | **<0.001** |
| Feeling free of desire | 0.02 | 0.02 | 1599.0 | 1.2 | 0.233 |
| Mind-wandering | -0.01 | 0.02 | 861.6 | -0.5 | 0.645 |
| **Surprise** | **0.04** | **0.02** | **1496.0** | **2.4** | **0.016** |
| **Wanting to understand more** | **0.08** | **0.02** | **1526.0** | **3.9** | **<0.001** |
| Telling a story | 0.03 | 0.02 | 1377.0 | 1.8 | 0.068 |

*Notes.* The reference level for image category was the beautiful art image category. Significant fixed effects and interactions are highlighted in bold. The model explained 74.8 % of the variance.

**Table S4. The linear-mixed effects model for Experiment 1a, including art education as additional factor.**

| Random effects | | | | | |
| --- | --- | --- | --- | --- | --- |
|  | Variance | *SD* |  |  |  |
| Participant | 0.05 | 0.22 |  |  |  |
| Image | 0.02 | 0.14 |  |  |  |
| Fixed effects | | | | | |
|  | Estimate | *SE* | *df* | *t* | *p* |
| **Intercept** | **0.30** | **0.11** | **163** | **2.78** | **0.006** |
| Art education | -0.03 | 0.25 | 405 | -0.13 | 0.901 |
| **Pleasure** | **0.26** | **0.02** | **1746** | **10.61** | **<0.001** |
| **Wish to continue** | **0.23** | **0.02** | **1629** | **10.24** | **<0.001** |
| **Feeling alive** | **0.13** | **0.02** | **1742** | **5.76** | **<0.001** |
| **Universality** | **0.20** | **0.02** | **1343** | **8.27** | **<0.001** |
| **Number of felt connections** | **0.07** | **0.02** | **1496** | **3.22** | **0.001** |
| Longing | 0.03 | 0.02 | 1354 | 1.54 | 0.124 |
| **Feeling free of desire** | **0.05** | **0.02** | **1491** | **2.35** | **0.019** |
| Mind-wandering | 0.03 | 0.02 | 1648 | 1.51 | 0.132 |
| Surprise | -0.02 | 0.02 | 1094 | -1.40 | 0.162 |
| Wanting to understand more | 0.02 | 0.02 | 1580 | 1.04 | 0.297 |
| Telling a story | -0.01 | 0.02 | 912 | -0.39 | 0.695 |
| Art education × pleasure | -0.03 | 0.07 | 1752 | -0.42 | 0.674 |
| Art education × Wish to continue | 0.00 | 0.08 | 1693 | 0.03 | 0.977 |
| Art education × Feeling alive | -0.05 | 0.07 | 1741 | -0.79 | 0.428 |
| **Art education × Universality** | -0.17 | 0.07 | 1685 | -2.37 | 0.018 |
| **Art education × Number of felt connections** | 0.14 | 0.07 | 1746 | 2.20 | 0.028 |
| Art education × Longing | 0.05 | 0.06 | 1636 | 0.90 | 0.368 |
| Art education × Feeling free of desire | 0.01 | 0.07 | 1687 | 0.11 | 0.915 |
| Art education × Mind-wandering | 0.05 | 0.06 | 1703 | 0.88 | 0.381 |
| Art education × Surprise | -0.05 | 0.05 | 1286 | -1.17 | 0.243 |
| **Art education × Wanting to understand more** | 0.00 | 0.06 | 1743 | 0.03 | 0.978 |
| Art education × Telling a story | 0.03 | 0.05 | 1744 | 0.64 | 0.525 |

*Notes.* The reference level for art education was no education. Significant fixed effects and interactions are highlighted in bold. The model explained 75.0 % of the variance.

**Table S5. The linear-mixed effects model for Experiment 1a, including philosophy education as additional factor.**

| Random effects | | | | | |
| --- | --- | --- | --- | --- | --- |
|  | Variance | *SD* |  |  |  |
| Participant | 0.05 | 0.21 |  |  |  |
| Image | 0.02 | 0.13 |  |  |  |
| Fixed effects | | | | | |
|  | Estimate | *SE* | *df* | *t* | *p* |
| **Intercept** | **0.30** | **0.11** | **157** | **2.81** | **0.006** |
| Philosophy education | -0.17 | 0.29 | 421 | -0.60 | 0.547 |
| **Pleasure** | **0.26** | **0.02** | **1739** | **10.84** | **<0.001** |
| **Wish to continue** | **0.23** | **0.02** | **1629** | **10.45** | **<0.001** |
| **Feeling alive** | **0.13** | **0.02** | **1742** | **5.71** | **0.000** |
| **Universality** | **0.19** | **0.02** | **1285** | **8.27** | **<0.001** |
| **Number of felt connections** | **0.07** | **0.02** | **1486** | **3.45** | **0.001** |
| Longing | 0.03 | 0.02 | 1332 | 1.76 | 0.078 |
| **Feeling free of desire** | **0.05** | **0.02** | **1468** | **2.41** | **0.016** |
| Mind-wandering | 0.03 | 0.02 | 1651 | 1.53 | 0.125 |
| Surprise | -0.02 | 0.02 | 1098 | -1.23 | 0.219 |
| **Wanting to understand more** | 0.02 | 0.02 | 1564 | 1.09 | 0.274 |
| Telling a story | -0.01 | 0.02 | 821 | -0.62 | 0.538 |
| Philosophy education × pleasure | -0.08 | 0.08 | 1708 | -1.08 | 0.278 |
| Philosophy education × Wish to continue | -0.08 | 0.09 | 1746 | -0.98 | 0.329 |
| Philosophy education × Feeling alive | 0.02 | 0.08 | 1738 | 0.23 | 0.820 |
| **Philosophy education × Universality** | **-0.17** | **0.08** | **1749** | **-2.06** | **0.039** |
| **Philosophy education × Number of felt connections** | **0.17** | **0.08** | **1743** | **2.20** | **0.028** |
| Philosophy education × Longing | 0.03 | 0.06 | 1674 | 0.46 | 0.643 |
| Philosophy education × Feeling free of desire | 0.03 | 0.07 | 1749 | 0.38 | 0.707 |
| Philosophy education × Mind-wandering | 0.05 | 0.07 | 1691 | 0.76 | 0.449 |
| Philosophy education × Surprise | -0.07 | 0.05 | 1356 | -1.35 | 0.179 |
| **Philosophy education × Wanting to understand more** | -0.01 | 0.07 | 1725 | -0.11 | 0.915 |
| Philosophy education × Telling a story | 0.11 | 0.06 | 1729 | 1.82 | 0.069 |

*Notes.* The reference level for philosophy education was no education. Significant fixed effects and interactions are highlighted in bold. The model explained 75.1 % of the variance.

**Table S6. The linear-mixed effects model for Experiment 1a, including age as additional predictor.**

| Random effects | | | | | |
| --- | --- | --- | --- | --- | --- |
|  | Variance | *SD* |  |  |  |
| Participant | 0.04 | 0.20 |  |  |  |
| Image | 0.02 | 0.15 |  |  |  |
| Fixed effects | | | | | |
|  | Estimate | *SE* | *df* | *t* | *p* |
| Intercept | -0.33 | 0.33 | 397 | -1.00 | 0.319 |
| **Age** | **0.02** | **0.01** | **369** | **2.11** | **0.036** |
| **Pleasure** | **0.42** | **0.09** | **1744** | **4.75** | **0.000** |
| **Wish to continue** | **0.32** | **0.09** | **1745** | **3.74** | **0.000** |
| Feeling alive | 0.06 | 0.08 | 1734 | 0.75 | 0.453 |
| Universality | 0.01 | 0.09 | 1609 | 0.11 | 0.916 |
| Number of felt connections | -0.02 | 0.07 | 1438 | -0.29 | 0.772 |
| **Longing** | **0.16** | **0.07** | **1377** | **2.34** | **0.019** |
| Feeling free of desire | 0.04 | 0.07 | 1385 | 0.65 | 0.516 |
| **Mind-wandering** | **0.21** | **0.07** | **1685** | **2.93** | **0.003** |
| **Surprise** | **-0.12** | **0.06** | **1007** | **-2.08** | **0.038** |
| **Wanting to understand more** | **0.15** | **0.07** | **1602** | **2.23** | **0.026** |
| **Telling a story** | **-0.19** | **0.06** | **1735** | **-3.18** | **0.002** |
| **Age × pleasure** | **0.00** | **0.00** | **1747** | **-1.89** | **0.059** |
| Age × Wish to continue | 0.00 | 0.00 | 1742 | -0.98 | 0.330 |
| Age × Feeling alive | 0.00 | 0.00 | 1745 | 0.76 | 0.450 |
| **Age × Universality** | **0.00** | **0.00** | **1654** | **1.98** | **0.048** |
| Age × Number of felt connections | 0.00 | 0.00 | 1495 | 1.39 | 0.165 |
| Age × Longing | 0.00 | 0.00 | 1413 | -1.95 | 0.051 |
| Age × Feeling free of desire | 0.00 | 0.00 | 1376 | -0.07 | 0.941 |
| **Age × Mind-wandering** | **0.00** | **0.00** | **1684** | **-2.48** | **0.013** |
| Age × Surprise | 0.00 | 0.00 | 1056 | 1.58 | 0.115 |
| Age × Wanting to understand more | 0.00 | 0.00 | 1599 | -1.88 | 0.060 |
| **Age × Telling a story** | **0.01** | **0.00** | **1740** | **3.25** | **0.001** |

*Notes.* Significant fixed effects and interactions are highlighted in bold. The model explained 75.2 % of the variance.

**Table S7. The linear-mixed effects model for Experiment 1a, 1a including political orientation as additional predictor.**

| Random effects | | | | | |
| --- | --- | --- | --- | --- | --- |
|  | Variance | *SD* |  |  |  |
| Participant | 0.04 | 0.20 |  |  |  |
| Image | 0.02 | 0.13 |  |  |  |
| Fixed effects | | | | | |
|  | Estimate | *SE* | *df* | *t* | *p* |
| Intercept | 0.30 | 0.17 | 393 | 1.82 | 0.069 |
| Conservativism | 0.01 | 0.04 | 521 | 0.28 | 0.780 |
| **Pleasure** | **0.20** | **0.05** | **1688** | **4.39** | **<0.001** |
| **Wish to continue** | **0.36** | **0.04** | **1620** | **8.97** | **<0.001** |
| Feeling alive | 0.04 | 0.04 | 1738 | 0.99 | 0.322 |
| **Universality** | **0.20** | **0.04** | **1443** | **4.57** | **<0.001** |
| **Number of felt connections** | **0.10** | **0.04** | **1527** | **2.56** | **0.011** |
| **Longing** | **0.08** | **0.03** | **1493** | **2.24** | **0.025** |
| Feeling free of desire | 0.01 | 0.04 | 1463 | 0.16 | 0.873 |
| **Mind-wandering** | **0.09** | **0.04** | **1666** | **2.31** | **0.021** |
| **Surprise** | **-0.09** | **0.03** | **1383** | **-2.73** | **0.006** |
| Wanting to understand more | 0.00 | 0.03 | 1742 | -0.11 | 0.915 |
| Telling a story | 0.00 | 0.03 | 1474 | -0.08 | 0.940 |
| Conservativism × pleasure | 0.02 | 0.01 | 1697 | 1.42 | 0.155 |
| **Conservativism × Wish to continue** | **-0.04** | **0.01** | **1634** | **-3.80** | **<0.001** |
| **Conservativism × Feeling alive** | **0.03** | **0.01** | **1748** | **2.17** | **0.030** |
| Conservativism × Universality | -0.01 | 0.01 | 1584 | -0.52 | 0.605 |
| Conservativism × Number of felt connections | 0.00 | 0.01 | 1553 | -0.42 | 0.674 |
| Conservativism × Longing | -0.01 | 0.01 | 1537 | -1.62 | 0.107 |
| Conservativism × Feeling free of desire | 0.01 | 0.01 | 1532 | 1.09 | 0.277 |
| Conservativism × Mind-wandering | -0.02 | 0.01 | 1638 | -1.69 | 0.092 |
| **Conservativism × Surprise** | **0.02** | **0.01** | **1404** | **2.14** | **0.033** |
| Conservativism × Wanting to understand more | 0.01 | 0.01 | 1749 | 1.20 | 0.232 |
| Conservativism × Telling a story | 0.00 | 0.01 | 1655 | -0.31 | 0.759 |

*Notes.* The reference level for image category was the beautiful art image category. Political orientation was measured on a strong liberal (1) to strong conservative (7) scale. Significant fixed effects and interactions are highlighted in bold. The model explained 75.2 % of the variance.

**Table S8. The linear-mixed effects model for Experiment 1a,  including the general beauty attitude question “How closely related are the feelings of beauty and pleasure?” as additional predictor.**

| Random effects | | | | | |
| --- | --- | --- | --- | --- | --- |
|  | Variance | *SD* |  |  |  |
| Participant | 0.05 | 0.22 |  |  |  |
| Image | 0.02 | 0.14 |  |  |  |
| Fixed effects | | | | | |
|  | Estimate | *SE* | *df* | *t* | *p* |
| Intercept | -0.08 | 0.36 | 388 | -0.22 | 0.823 |
| Closeness beauty pleasure (CBP) | 0.07 | 0.06 | 399 | 1.14 | 0.256 |
| **Pleasure** | **0.46** | **0.10** | **1722** | **4.38** | **<0.001** |
| **Wish to continue** | **0.38** | **0.08** | **1633** | **4.53** | **<0.001** |
| Feeling alive | 0.08 | 0.10 | 1728 | 0.82 | 0.413 |
| Universality | 0.11 | 0.09 | 1497 | 1.14 | 0.253 |
| Number of felt connections | -0.04 | 0.08 | 1145 | -0.55 | 0.585 |
| Longing | -0.02 | 0.08 | 1425 | -0.21 | 0.836 |
| Feeling free of desire | 0.06 | 0.08 | 1673 | 0.66 | 0.508 |
| Mind-wandering | 0.01 | 0.09 | 1657 | 0.13 | 0.897 |
| Surprise | 0.08 | 0.08 | 1268 | 0.98 | 0.326 |
| Wanting to understand more | -0.05 | 0.08 | 1737 | -0.63 | 0.530 |
| Telling a story | 0.02 | 0.07 | 1244 | 0.24 | 0.810 |
| **CBP × pleasure** | **-0.04** | **0.02** | **1730** | **-2.01** | **0.045** |
| CBP × Wish to continue | -0.03 | 0.02 | 1619 | -1.82 | 0.070 |
| CBP × Feeling alive | 0.01 | 0.02 | 1729 | 0.48 | 0.628 |
| CBP × Universality | 0.01 | 0.02 | 1454 | 0.82 | 0.412 |
| CBP × Number of felt connections | 0.02 | 0.01 | 1270 | 1.59 | 0.111 |
| CBP × Longing | 0.01 | 0.01 | 1493 | 0.67 | 0.501 |
| CBP × Feeling free of desire | 0.00 | 0.02 | 1652 | -0.14 | 0.890 |
| CBP × Mind-wandering | 0.00 | 0.02 | 1664 | 0.27 | 0.787 |
| CBP × Surprise | -0.02 | 0.01 | 1307 | -1.37 | 0.172 |
| CBP × Wanting to understand more | 0.01 | 0.01 | 1745 | 0.91 | 0.361 |
| CBP × Telling a story | 0.00 | 0.01 | 1349 | -0.30 | 0.765 |

*Notes.* Significant fixed effects and interactions are highlighted in bold. The model explained 75.0 % of the variance.

**Table S9. The linear-mixed effects model for Experiment 1a, including the general beauty attitude question “Which is greater, the beauty of art or nature?” as additional predictor.**

| Random effects | | | | | |
| --- | --- | --- | --- | --- | --- |
|  | Variance | *SD* |  |  |  |
| Participant | 0.04 | 0.21 |  |  |  |
| Image | 0.02 | 0.14 |  |  |  |
| Fixed effects | | | | | |
|  | Estimate | *SE* | *df* | *t* | *p* |
| Intercept | 0.58 | 0.33 | 424 | 1.73 | 0.084 |
| Art or nature | -0.04 | 0.06 | 418 | -0.76 | 0.449 |
| Pleasure | 0.09 | 0.09 | 1744 | 0.98 | 0.325 |
| **Wish to continue** | **0.19** | **0.08** | **1713** | **2.31** | **0.021** |
| Feeling alive | 0.16 | 0.08 | 1749 | 1.95 | 0.051 |
| Universality | 0.10 | 0.08 | 1704 | 1.21 | 0.228 |
| **Number of felt connections** | **0.23** | **0.08** | **1489** | **2.90** | **0.004** |
| Longing | 0.08 | 0.07 | 1426 | 1.21 | 0.226 |
| Feeling free of desire | 0.05 | 0.08 | 1541 | 0.69 | 0.493 |
| Mind-wandering | 0.09 | 0.08 | 1625 | 1.11 | 0.270 |
| Surprise | 0.00 | 0.06 | 1196 | -0.08 | 0.940 |
| Wanting to understand more | -0.03 | 0.07 | 1635 | -0.45 | 0.653 |
| Telling a story | -0.01 | 0.06 | 1538 | -0.19 | 0.846 |
| **Art or nature × pleasure** | **0.03** | **0.02** | **1742** | **2.05** | **0.041** |
| Art or nature × Wish to continue | 0.01 | 0.01 | 1713 | 0.58 | 0.560 |
| Art or nature × Feeling alive | -0.01 | 0.01 | 1741 | -0.44 | 0.662 |
| Art or nature × Universality | 0.02 | 0.01 | 1707 | 1.00 | 0.317 |
| **Art or nature × Number of felt connections** | **-0.03** | **0.01** | **1473** | **-1.98** | **0.048** |
| Art or nature × Longing | -0.01 | 0.01 | 1427 | -0.79 | 0.429 |
| Art or nature × Feeling free of desire | 0.00 | 0.01 | 1535 | -0.11 | 0.916 |
| Art or nature × Mind-wandering | -0.01 | 0.01 | 1638 | -0.68 | 0.500 |
| Art or nature × Surprise | 0.00 | 0.01 | 1247 | -0.44 | 0.658 |
| **Art or nature × Wanting to understand more** | 0.01 | 0.01 | 1671 | 0.78 | 0.438 |
| Art or nature × Telling a story | 0.00 | 0.01 | 1562 | 0.08 | 0.934 |

*Notes.* The reference level for image category was the beautiful art image category. Significant fixed effects and interactions are highlighted in bold. The model explained 74.9 % of the variance.

**Table S10. The linear-mixed effects model for Experiment 1a, including the general beauty attitude question “The beauty of an image is…” entirely in the image (1) to entirely in the story (7) as additional predictor.**

| Random effects | | | | | |
| --- | --- | --- | --- | --- | --- |
|  | Variance | *SD* |  |  |  |
| Participant | 0.05 | 0.21 |  |  |  |
| Image | 0.02 | 0.14 |  |  |  |
| Fixed effects | | | | | |
|  | Estimate | *SE* | *df* | *t* | *p* |
| **Intercept** | **0.69** | **0.28** | **481** | **2.42** | **0.016** |
| Image vs story | -0.09 | 0.07 | 481 | -1.33 | 0.186 |
| **Pleasure** | **0.30** | **0.08** | **1748** | **3.97** | **<0.001** |
| **Wish to continue** | **0.32** | **0.07** | **1748** | **4.66** | **<0.001** |
| Feeling alive | 0.11 | 0.08 | 1742 | 1.44 | 0.150 |
| Universality | 0.11 | 0.07 | 1619 | 1.56 | 0.119 |
| Number of felt connections | 0.04 | 0.07 | 1470 | 0.66 | 0.508 |
| Longing | 0.01 | 0.06 | 1347 | 0.23 | 0.820 |
| Feeling free of desire | 0.03 | 0.07 | 1692 | 0.49 | 0.627 |
| Mind-wandering | 0.04 | 0.07 | 1634 | 0.56 | 0.576 |
| Surprise | -0.04 | 0.06 | 1595 | -0.69 | 0.488 |
| Wanting to understand more | 0.09 | 0.07 | 1747 | 1.38 | 0.168 |
| **Telling a story** | **-0.12** | **0.06** | **1515** | **-1.97** | **0.049** |
| Image vs story × pleasure | -0.01 | 0.02 | 1740 | -0.65 | 0.518 |
| Image vs story × Wish to continue | -0.02 | 0.02 | 1752 | -1.40 | 0.162 |
| Image vs story × Feeling alive | 0.01 | 0.02 | 1734 | 0.34 | 0.737 |
| Image vs story × Universality | 0.02 | 0.02 | 1713 | 0.98 | 0.327 |
| Image vs story × Number of felt connections | 0.01 | 0.02 | 1553 | 0.58 | 0.562 |
| Image vs story × Longing | 0.00 | 0.02 | 1399 | 0.33 | 0.740 |
| Image vs story × Feeling free of desire | 0.00 | 0.02 | 1657 | 0.12 | 0.901 |
| Image vs story × Mind-wandering | 0.00 | 0.02 | 1665 | -0.12 | 0.903 |
| Image vs story × Surprise | 0.00 | 0.01 | 1636 | 0.19 | 0.853 |
| Image vs story × Telling a story | 0.03 | 0.01 | 1557 | 1.95 | 0.052 |

*Notes.* Significant fixed effects and interactions are highlighted in bold. The model explained 74.9 % of the variance.

**Table S11. The linear-mixed effects model for Experiment 1a, including the general beauty attitude question “Are shared experiences of beauty a form of communication?” as additional predictor.**

| Random effects | | | | | |
| --- | --- | --- | --- | --- | --- |
|  | Variance | *SD* |  |  |  |
| Participant | 0.04 | 0.21 |  |  |  |
| Image | 0.02 | 0.14 |  |  |  |
| Fixed effects | | | | | |
|  | Estimate | *SE* | *df* | *t* | *p* |
| Intercept | 0.27 | 0.31 | 347 | 0.90 | 0.371 |
| Communication | 0.02 | 0.05 | 358 | 0.29 | 0.774 |
| **Pleasure** | **0.27** | **0.09** | **1745** | **2.86** | **0.004** |
| **Wish to continue** | **0.31** | **0.07** | **1588** | **4.18** | **<0.001** |
| Feeling alive | 0.03 | 0.09 | 1735 | 0.32 | 0.749 |
| Universality | 0.09 | 0.09 | 1692 | 1.08 | 0.281 |
| **Number of felt connections** | **0.16** | **0.08** | **1130** | **2.10** | **0.036** |
| Longing | 0.13 | 0.07 | 1527 | 1.78 | 0.076 |
| **Feeling free of desire** | **0.17** | **0.08** | **1700** | **2.09** | **0.036** |
| Mind-wandering | -0.04 | 0.08 | 1649 | -0.55 | 0.585 |
| Surprise | -0.02 | 0.07 | 1072 | -0.26 | 0.798 |
| **Wanting to understand more** | -0.04 | 0.07 | 1683 | -0.61 | 0.545 |
| Telling a story | -0.08 | 0.06 | 1369 | -1.27 | 0.206 |
| Communication × pleasure | 0.00 | 0.02 | 1747 | -0.08 | 0.939 |
| Communication × Wish to continue | -0.01 | 0.01 | 1609 | -1.06 | 0.288 |
| Communication × Feeling alive | 0.02 | 0.02 | 1737 | 1.15 | 0.249 |
| Communication × Universality | 0.02 | 0.02 | 1677 | 1.01 | 0.314 |
| Communication × Number of felt connections | -0.02 | 0.01 | 1172 | -1.13 | 0.258 |
| Communication × Longing | -0.02 | 0.01 | 1473 | -1.41 | 0.159 |
| Communication × Feeling free of desire | -0.02 | 0.01 | 1624 | -1.56 | 0.118 |
| Communication × Mind-wandering | 0.01 | 0.01 | 1647 | 0.98 | 0.326 |
| Communication × Surprise | 0.00 | 0.01 | 1087 | -0.22 | 0.827 |
| Communication × Wanting to understand more | 0.01 | 0.01 | 1671 | 1.02 | 0.310 |
| Communication × Telling a story | 0.01 | 0.01 | 1333 | 1.14 | 0.257 |

*Notes.* The reference level for image category was the beautiful art image category. Significant fixed effects and interactions are highlighted in bold. The model explained 74.9 % of the variance.

**Table S12. The linear-mixed effects model for Experiment 1a, including the general beauty attitude question “How much does mood affect the feeling of beauty?” as additional predictor.**

| Random effects | | | | | |
| --- | --- | --- | --- | --- | --- |
|  | Variance | *SD* |  |  |  |
| Participant | 0.04 | 0.21 |  |  |  |
| Image | 0.02 | 0.14 |  |  |  |
| Fixed effects | | | | | |
|  | Estimate | *SE* | *df* | *t* | *p* |
| Intercept | 0.02 | 0.40 | 572 | 0.05 | 0.964 |
| Mood | 0.05 | 0.07 | 555 | 0.82 | 0.415 |
| Pleasure | 0.04 | 0.12 | 1750 | 0.33 | 0.740 |
| Wish to continue | 0.13 | 0.11 | 1675 | 1.19 | 0.234 |
| **Feeling alive** | **0.29** | **0.11** | **1747** | **2.69** | **0.007** |
| **Universality** | **0.23** | **0.10** | **1712** | **2.21** | **0.028** |
| Number of felt connections | 0.12 | 0.09 | 1418 | 1.33 | 0.184 |
| Longing | 0.13 | 0.09 | 1664 | 1.45 | 0.148 |
| Feeling free of desire | 0.13 | 0.10 | 1606 | 1.35 | 0.178 |
| Mind-wandering | -0.01 | 0.10 | 1738 | -0.15 | 0.880 |
| Surprise | 0.04 | 0.08 | 1114 | 0.48 | 0.630 |
| Wanting to understand more | 0.02 | 0.09 | 1666 | 0.17 | 0.867 |
| Telling a story | -0.06 | 0.08 | 1565 | -0.79 | 0.431 |
| Mood × pleasure | 0.04 | 0.02 | 1751 | 1.78 | 0.075 |
| Mood × Wish to continue | 0.02 | 0.02 | 1676 | 0.93 | 0.353 |
| Mood × Feeling alive | -0.03 | 0.02 | 1747 | -1.55 | 0.122 |
| Mood × Universality | -0.01 | 0.02 | 1661 | -0.39 | 0.693 |
| Mood × Number of felt connections | -0.01 | 0.02 | 1411 | -0.55 | 0.583 |
| Mood × Longing | -0.02 | 0.01 | 1646 | -1.13 | 0.260 |
| Mood × Feeling free of desire | -0.01 | 0.02 | 1584 | -0.88 | 0.378 |
| Mood × Mind-wandering | 0.01 | 0.02 | 1737 | 0.51 | 0.611 |
| Mood × Surprise | -0.01 | 0.01 | 1065 | -0.87 | 0.383 |
| Mood × Wanting to understand more | 0.00 | 0.02 | 1665 | 0.09 | 0.930 |
| Mood × Telling a story | 0.01 | 0.01 | 1562 | 0.65 | 0.517 |

*Notes.* The reference level for image category was the beautiful art image category. Significant fixed effects and interactions are highlighted in bold. The model explained 74.8 % of the variance.

**Table S13. The linear-mixed effects model for Experiment 1a, including the general beauty attitude question “Can you name an object that everyone finds beautiful?” as additional predictor.**

| Random effects | | | | | |
| --- | --- | --- | --- | --- | --- |
|  | Variance | *SD* |  |  |  |
| Participant | 0.04 | 0.21 |  |  |  |
| Image | 0.02 | 0.13 |  |  |  |
| Fixed effects | | | | | |
|  | Estimate | *SE* | *df* | *t* | *p* |
| Intercept | 0.17 | 0.14 | 293 | 1.24 | 0.217 |
| Universally beautiful object (UBO) | 0.24 | 0.17 | 596 | 1.40 | 0.163 |
| **Pleasure** | **0.23** | **0.03** | **1753** | **6.88** | **<0.001** |
| **Wish to continue** | **0.21** | **0.03** | **1710** | **6.26** | **<0.001** |
| **Feeling alive** | **0.11** | **0.03** | **1747** | **3.36** | **0.001** |
| **Universality** | **0.22** | **0.03** | **1693** | **6.50** | **<0.001** |
| **Number of felt connections** | **0.10** | **0.03** | **1566** | **3.40** | **0.001** |
| Longing | 0.04 | 0.03 | 1322 | 1.52 | 0.130 |
| Feeling free of desire | 0.03 | 0.03 | 1371 | 1.25 | 0.213 |
| Mind-wandering | 0.03 | 0.03 | 1656 | 0.92 | 0.358 |
| Surprise | -0.03 | 0.02 | 1094 | -1.17 | 0.243 |
| Wanting to understand more | 0.03 | 0.03 | 1693 | 1.16 | 0.245 |
| Telling a story | 0.01 | 0.03 | 1518 | 0.23 | 0.817 |
| UBO × pleasure | 0.05 | 0.05 | 1744 | 1.00 | 0.315 |
| UBO × Wish to continue | 0.04 | 0.04 | 1660 | 0.92 | 0.358 |
| UBO × Feeling alive | 0.03 | 0.04 | 1736 | 0.69 | 0.491 |
| UBO × Universality | -0.07 | 0.04 | 1572 | -1.57 | 0.116 |
| UBO × Number of felt connections | -0.04 | 0.04 | 1506 | -0.97 | 0.334 |
| UBO × Longing | -0.02 | 0.04 | 1318 | -0.53 | 0.598 |
| UBO × Feeling free of desire | 0.02 | 0.04 | 1454 | 0.54 | 0.592 |
| UBO × Mind-wandering | 0.01 | 0.04 | 1652 | 0.17 | 0.864 |
| UBO × Surprise | -0.01 | 0.03 | 1138 | -0.18 | 0.854 |
| UBO × Wanting to understand more | -0.02 | 0.04 | 1694 | -0.55 | 0.586 |
| UBO × Telling a story | -0.02 | 0.03 | 1643 | -0.56 | 0.574 |

*Notes.* The reference level for universal beauty was “yes”. Significant fixed effects and interactions are highlighted in bold. The model explained 74.8 % of the variance.

## Comparison to data from Menninghaus et al. (2019)

Since Menninghaus and colleagues (2019a) also employed an unpleasant-pleasant scale in their study asking people about their conception of beauty with the same 1-7 range we used, and their data is openly accessible, we compared the ratings given by our and their participants to increase the generalizability of our results. From our data, we used trials of stimuli with a beauty rating of 6 or 7. Pleasure ratings for the concept beauty in Menninghaus and colleagues’ study and our data for beautiful images were no different, *p* = 0.325, *MD* = 0.14, 95% CI [-0.14, 0.43]. This underlines the stable association between high pleasure and beauty.

# Experiment 1b: separate linear models

Of all linear mixed-effects models tested (see **Supplemental Tables S14-S22**), one that incorporated random effects of participant and stimulus, as well as the interaction between stimulus category and the eleven non-beauty ratings, predicted beauty ratings best, *R*^2^ = 0.73, performance 81.64%, all remaining performances ≤ 80.88%. The model effects for this best model are listed in **Supplemental Table S14**. Beauty ratings were, on average, 1.23 points lower for songs than for images. Beauty generally increased with increasing ratings of pleasure, Wish to continue, a feeling that the image is beautiful to everyone, feeling free of desire, and mind-wandering. The increase of beauty with increasing mind-wandering was the only effect observed in Experiment 1b but not Experiment 1a. In addition, we saw a greater increase in beauty with an increasing wish to continue the experience for images. In contrast, beauty increased more with increasing ratings of that stimulus being beautiful for everyone for songs compared to images. Ratings of longing and of the extent to which the stimulus tells a story are related positively with beauty ratings for songs only, not for images.

**Table S14. The mixed-effects model that best predicts rated beauty in Experiment 1b.**

| Random effects | | | | | |
| --- | --- | --- | --- | --- | --- |
|  | Variance | *SD* |  |  |  |
| Participant | 0.10 | 0.31 |  |  |  |
| Stimulus | 0.02 | 0.14 |  |  |  |
| Fixed effects | | | | | |
|  | Estimate | *SE* | *df* | *t* | *p* |
| **(Intercept)** | **0.93** | **0.28** | **385.0** | **3.32** | **0.001** |
| **Song** | **-1.23** | **0.34** | **266.2** | **-3.63** | **<0.001** |
| **Pleasure*** | **0.21** | **0.05** | **822.1** | **4.40** | **<0.001** |
| **Wish to continue*** | **0.26** | **0.05** | **848.2** | **5.49** | **<0.001** |
| Feeling alive | 0.07 | 0.05 | 854.6 | 1.55 | 0.121 |
| **Universality*** | **0.13** | **0.05** | **799.6** | **2.78** | **0.006** |
| Number of felt connections | 0.03 | 0.04 | 822.5 | 0.59 | 0.557 |
| Longing | -0.03 | 0.04 | 808.8 | -0.88 | 0.377 |
| **Feeling free of desire*** | **0.14** | **0.04** | **803.5** | **3.50** | **<0.001** |
| **Mind-wandering** | **0.10** | **0.04** | **788.2** | **2.37** | **0.018** |
| Surprise | 0.00 | 0.03 | 800.3 | 0.04 | 0.969 |
| Wanting to understand more | 0.03 | 0.04 | 821.7 | 0.70 | 0.483 |
| Telling a story | -0.07 | 0.04 | 799.8 | -1.79 | 0.075 |
| Song × pleasure | -0.05 | 0.05 | 852.1 | -1.00 | 0.320 |
| **Song × Wish to continue** | **0.20** | **0.06** | **834.3** | **3.30** | **0.001** |
| Song × feeling alive | -0.12 | 0.06 | 845.3 | -1.89 | 0.060 |
| **Song × Universality** | **-0.19** | **0.06** | **855.6** | **-3.01** | **0.003** |
| Song × number of felt connections | 0.05 | 0.06 | 857.6 | 0.98 | 0.329 |
| Song × longing | -0.08 | 0.05 | 835.4 | -1.40 | 0.163 |
| Song × feeling free of desire | 0.06 | 0.05 | 850.1 | 1.31 | 0.192 |
| Song × mind-wandering | -0.07 | 0.06 | 790.6 | -1.23 | 0.219 |
| Song × surprise | 0.05 | 0.06 | 848.1 | 0.78 | 0.436 |
| **Song × wanting to understand more** | **0.17** | **0.05** | **838.5** | **3.28** | **0.001** |
| **Song × telling a story** | **0.15** | **0.05** | **692.6** | **2.89** | **0.004** |

*Notes.* The reference level for stimulus category was image. Significant fixed effects and interactions are highlighted in bold. The model explained 73 % of the variance. *Main effect is present in Experiment 1a and 1b.

**Table S15. The linear-mixed effects model for Experiment 1b, including gender as additional factor.**

| Random effects | | | | | |
| --- | --- | --- | --- | --- | --- |
|  | Variance | *SD* |  |  |  |
| Participant | 0.1 | 0.32 |  |  |  |
| Image | 0.2 | 0.47 |  |  |  |
| Fixed effects | | | | | |
|  | Estimate | *SE* | *df* | *t* | *p* |
| Intercept | -0.13 | 0.51 | 402.4 | -0.25 | 0.804 |
| Gender | 0.35 | 0.31 | 559.4 | 1.13 | 0.258 |
| Pleasure | 0.14 | 0.11 | 825.9 | 1.32 | 0.186 |
| **Wish to continue** | **0.22** | **0.10** | **849.9** | **2.26** | **0.024** |
| Feeling alive | 0.08 | 0.09 | 849.7 | 0.89 | 0.372 |
| **Universality** | **0.30** | **0.09** | **790.8** | **3.35** | **0.001** |
| Number of felt connections | 0.05 | 0.10 | 778.1 | 0.48 | 0.630 |
| Longing | -0.13 | 0.09 | 641.0 | -1.47 | 0.143 |
| Feeling free of desire | 0.03 | 0.08 | 843.9 | 0.36 | 0.718 |
| **Mind-wandering** | **0.35** | **0.08** | **760.0** | **4.29** | **<0.001** |
| Surprise | -0.05 | 0.08 | 753.5 | -0.69 | 0.491 |
| Wanting to understand more | 0.03 | 0.09 | 832.7 | 0.33 | 0.745 |
| Telling a story | -0.01 | 0.08 | 852.1 | -0.12 | 0.905 |
| Gender × pleasure | 0.00 | 0.07 | 803.1 | -0.01 | 0.990 |
| Gender × Wish to continue | -0.02 | 0.06 | 847.9 | -0.38 | 0.701 |
| Gender × Feeling alive | -0.04 | 0.06 | 848.5 | -0.74 | 0.461 |
| Gender × Universality | -0.02 | 0.06 | 780.4 | -0.35 | 0.727 |
| Gender × Number of felt connections | 0.01 | 0.07 | 774.4 | 0.14 | 0.891 |
| Gender × Longing | 0.11 | 0.06 | 729.8 | 1.93 | 0.055 |
| Gender × Feeling free of desire | 0.05 | 0.05 | 845.2 | 0.88 | 0.382 |
| **Gender × Mind-wandering** | **-0.19** | **0.05** | **768.2** | **-3.55** | **<0.001** |
| Gender × Surprise | 0.05 | 0.05 | 737.4 | 1.01 | 0.312 |
| Gender × Wanting to understand more | 0.02 | 0.06 | 831.2 | 0.41 | 0.681 |
| Gender × Telling a story | -0.01 | 0.05 | 848.4 | -0.28 | 0.782 |

*Notes.* The reference level for gender was male. Significant fixed effects and interactions are highlighted in bold. The model explained 68 % of the variance.

**Table S16. The linear-mixed effects model for Experiment 1b, including age as additional predictor.**

| Random effects | | | | | |
| --- | --- | --- | --- | --- | --- |
|  | Variance | *SD* |  |  |  |
| Participant | 0.1 | 0.31 |  |  |  |
| Image | 0.2 | 0.44 |  |  |  |
| Fixed effects | | | | | |
|  | Estimate | *SE* | *df* | *t* | *p* |
| Intercept | 0.06 | 0.57 | 413.9 | 0.10 | 0.921 |
| Age | 0.01 | 0.01 | 408.6 | 0.52 | 0.606 |
| Pleasure | 0.15 | 0.12 | 837.5 | 1.25 | 0.213 |
| **Wish to continue** | **0.34** | **0.10** | **853.0** | **3.20** | **0.001** |
| Feeling alive | 0.06 | 0.10 | 848.5 | 0.57 | 0.569 |
| **Universality** | **0.62** | **0.11** | **765.0** | **5.87** | **<0.001** |
| Number of felt connections | -0.19 | 0.11 | 734.0 | -1.76 | 0.079 |
| Longing | 0.06 | 0.10 | 789.4 | 0.61 | 0.541 |
| Feeling free of desire | 0.01 | 0.10 | 849.6 | 0.09 | 0.925 |
| Mind-wandering | -0.07 | 0.10 | 822.1 | -0.71 | 0.481 |
| Surprise | 0.02 | 0.09 | 772.1 | 0.19 | 0.848 |
| Wanting to understand more | -0.09 | 0.10 | 843.0 | -0.88 | 0.380 |
| Telling a story | 0.04 | 0.09 | 848.5 | 0.47 | 0.636 |
| Age × pleasure | 0.00 | 0.00 | 838.9 | -0.19 | 0.848 |
| Age × Wish to continue | 0.00 | 0.00 | 849.8 | -1.55 | 0.122 |
| Age × Feeling alive | 0.00 | 0.00 | 848.2 | -0.46 | 0.644 |
| **Age × Universality** | **-0.01** | **0.00** | **682.2** | **-3.33** | **0.001** |
| **Age × Number of felt connections** | **0.01** | **0.00** | **755.8** | **2.39** | **0.017** |
| Age × Longing | 0.00 | 0.00 | 770.6 | -0.27 | 0.787 |
| Age × Feeling free of desire | 0.00 | 0.00 | 849.6 | 0.88 | 0.381 |
| Age × Mind-wandering | 0.00 | 0.00 | 840.4 | 1.56 | 0.118 |
| Age × Surprise | 0.00 | 0.00 | 769.8 | 0.13 | 0.895 |
| Age × Wanting to understand more | 0.00 | 0.00 | 849.9 | 1.65 | 0.100 |
| Age × Telling a story | 0.00 | 0.00 | 850.7 | -0.83 | 0.406 |

*Notes.* Significant fixed effects and interactions are highlighted in bold. The model explained 69 % of the variance.

**Table S17. The linear-mixed effects model for Experiment 1b, including the general beauty attitude question “How closely related are the feelings of beauty and pleasure?” as additional predictor.**

| Random effects | | | | | |
| --- | --- | --- | --- | --- | --- |
|  | Variance | *SD* |  |  |  |
| Participant | 0.11 | 0.33 |  |  |  |
| Image | 0.21 | 0.46 |  |  |  |
| Fixed effects | | | | | |
|  | Estimate | *SE* | *df* | *t* | *p* |
| Intercept | 0.36 | 0.73 | 506 | 0.50 | 0.621 |
| Closeness beauty pleasure (CBP) | 0.00 | 0.12 | 506 | -0.03 | 0.978 |
| **Pleasure** | **0.45** | **0.15** | **718** | **2.92** | **0.004** |
| **Wish to continue** | **0.34** | **0.12** | **844** | **2.73** | **0.007** |
| Feeling alive | 0.10 | 0.14 | 840 | 0.72 | 0.473 |
| Universality | 0.21 | 0.12 | 460 | 1.76 | 0.079 |
| Number of felt connections | -0.27 | 0.15 | 800 | -1.76 | 0.078 |
| Longing | 0.12 | 0.13 | 772 | 0.96 | 0.339 |
| Feeling free of desire | 0.10 | 0.12 | 849 | 0.84 | 0.401 |
| Mind-wandering | -0.13 | 0.12 | 792 | -1.09 | 0.277 |
| Surprise | -0.08 | 0.13 | 791 | -0.57 | 0.568 |
| Wanting to understand more | 0.14 | 0.13 | 837 | 1.06 | 0.289 |
| Telling a story | -0.03 | 0.11 | 852 | -0.26 | 0.798 |
| **CBP × pleasure** | **-0.06** | **0.03** | **730** | **-2.14** | **0.032** |
| CBP × Wish to continue | -0.03 | 0.02 | 845 | -1.33 | 0.182 |
| CBP × Feeling alive | -0.01 | 0.02 | 839 | -0.59 | 0.557 |
| CBP × Universality | 0.01 | 0.02 | 494 | 0.58 | 0.561 |
| **CBP × Number of felt connections** | **0.06** | **0.03** | **802** | **2.22** | **0.027** |
| CBP × Longing | -0.02 | 0.02 | 777 | -0.69 | 0.492 |
| CBP × Feeling free of desire | 0.00 | 0.02 | 848 | 0.00 | 0.999 |
| CBP × Mind-wandering | 0.04 | 0.02 | 800 | 1.86 | 0.063 |
| CBP × Surprise | 0.02 | 0.02 | 790 | 0.76 | 0.450 |
| CBP × Wanting to understand more | -0.01 | 0.02 | 846 | -0.56 | 0.575 |
| CBP × Telling a story | 0.00 | 0.02 | 849 | 0.00 | 0.998 |

*Notes.* Significant fixed effects and interactions are highlighted in bold. The model explained 69 % of the variance.

**Table S18. The linear-mixed effects model for Experiment 1b, including the general beauty attitude question “Which is greater, the beauty of art or nature?” as additional predictor.**

| Random effects | | | | | |
| --- | --- | --- | --- | --- | --- |
|  | Variance | *SD* |  |  |  |
| Participant | 0.10 | 0.32 |  |  |  |
| Image | 0.23 | 0.48 |  |  |  |
| Fixed effects | | | | | |
|  | Estimate | *SE* | *df* | *t* | *p* |
| Intercept | 0.66 | 0.75 | 473.7 | 0.88 | 0.382 |
| Art or nature | -0.04 | 0.13 | 479.1 | -0.30 | 0.768 |
| Pleasure | 0.23 | 0.15 | 772.4 | 1.51 | 0.132 |
| **Wish to continue** | **0.34** | **0.14** | **845.1** | **2.52** | **0.012** |
| Feeling alive | 0.18 | 0.14 | 848.7 | 1.28 | 0.200 |
| **Universality** | **0.32** | **0.12** | **828.3** | **2.58** | **0.010** |
| **Number of felt connections** | **-0.45** | **0.15** | **774.1** | **-2.90** | **0.004** |
| Longing | -0.03 | 0.13 | 817.5 | -0.21 | 0.836 |
| Feeling free of desire | 0.11 | 0.12 | 832.1 | 0.94 | 0.347 |
| Mind-wandering | -0.13 | 0.12 | 545.3 | -1.07 | 0.285 |
| Surprise | -0.12 | 0.12 | 612.0 | -1.05 | 0.292 |
| Wanting to understand more | 0.20 | 0.13 | 848.3 | 1.47 | 0.142 |
| Telling a story | 0.05 | 0.11 | 788.3 | 0.49 | 0.623 |
| Art or nature × pleasure | -0.02 | 0.03 | 800.6 | -0.59 | 0.555 |
| Art or nature × Wish to continue | -0.03 | 0.02 | 846.0 | -1.18 | 0.240 |
| Art or nature × Feeling alive | -0.03 | 0.02 | 847.3 | -1.25 | 0.210 |
| Art or nature × Universality | -0.01 | 0.02 | 826.4 | -0.46 | 0.648 |
| **Art or nature × Number of felt connections** | **0.09** | **0.03** | **760.3** | **3.38** | **0.001** |
| Art or nature × Longing | 0.01 | 0.02 | 809.6 | 0.48 | 0.633 |
| Art or nature × Feeling free of desire | 0.00 | 0.02 | 835.9 | -0.14 | 0.886 |
| Art or nature × Mind-wandering | 0.04 | 0.02 | 585.8 | 1.78 | 0.075 |
| Art or nature × Surprise | 0.02 | 0.02 | 635.4 | 1.17 | 0.245 |
| Art or nature × Wanting to understand more | -0.02 | 0.02 | 847.7 | -0.97 | 0.332 |
| Art or nature × Telling a story | -0.01 | 0.02 | 813.5 | -0.68 | 0.494 |

*Notes.* The reference level for image category was the beautiful art image category. Significant fixed effects and interactions are highlighted in bold. The model explained 69 % of the variance.

**Table S19. The linear-mixed effects model for Experiment 1b, including the general beauty attitude question “The beauty of an image is…” entirely in the image (1) to entirely in the story (7) as additional predictor.**

| Random effects | | | | | |
| --- | --- | --- | --- | --- | --- |
|  | Variance | *SD* |  |  |  |
| Participant | 0.10 | 0.31 |  |  |  |
| Image | 0.23 | 0.48 |  |  |  |
| Fixed effects | | | | | |
|  | Estimate | *SE* | *df* | *t* | *p* |
| Intercept | 0.19 | 0.59 | 521.1 | 0.33 | 0.742 |
| Image vs story | 0.05 | 0.13 | 691.6 | 0.35 | 0.728 |
| **Pleasure** | **0.49** | **0.12** | **818.8** | **4.19** | **<0.001** |
| **Wish to continue** | **0.29** | **0.10** | **845.4** | **2.76** | **0.006** |
| Feeling alive | 0.15 | 0.10 | 852.4 | 1.56 | 0.118 |
| Universality | 0.08 | 0.11 | 753.3 | 0.80 | 0.424 |
| Number of felt connections | -0.15 | 0.11 | 770.6 | -1.33 | 0.182 |
| Longing | 0.02 | 0.10 | 633.1 | 0.18 | 0.857 |
| Feeling free of desire | 0.06 | 0.09 | 850.3 | 0.68 | 0.494 |
| Mind-wandering | 0.13 | 0.09 | 790.0 | 1.35 | 0.178 |
| Surprise | -0.14 | 0.09 | 783.4 | -1.64 | 0.101 |
| Wanting to understand more | -0.02 | 0.09 | 835.1 | -0.23 | 0.817 |
| Telling a story | 0.05 | 0.08 | 852.7 | 0.57 | 0.569 |
| **Image vs story × pleasure** | **-0.09** | **0.03** | **839.8** | **-3.23** | **0.001** |
| Image vs story × Wish to continue | -0.03 | 0.02 | 842.4 | -1.11 | 0.269 |
| Image vs story × Feeling alive | -0.04 | 0.02 | 848.2 | -1.54 | 0.123 |
| Image vs story × Universality | 0.05 | 0.02 | 794.6 | 1.85 | 0.065 |
| **Image vs story × Number of felt connections** | **0.05** | **0.03** | **771.7** | **2.03** | **0.043** |
| Image vs story × Longing | 0.01 | 0.02 | 704.0 | 0.35 | 0.727 |
| Image vs story × Feeling free of desire | 0.01 | 0.02 | 849.1 | 0.31 | 0.760 |
| Image vs story × Mind-wandering | -0.01 | 0.02 | 819.6 | -0.46 | 0.644 |
| Image vs story × Surprise | 0.04 | 0.02 | 777.9 | 1.92 | 0.056 |
| Image vs story × Wanting to understand more | 0.02 | 0.02 | 840.0 | 0.97 | 0.334 |
| Image vs story × Telling a story | -0.02 | 0.02 | 845.0 | -0.83 | 0.409 |

*Notes.* Significant fixed effects and interactions are highlighted in bold. The model explained 69 % of the variance.

**Table S20. The linear-mixed effects model for Experiment 1b, including the general beauty attitude question “Are shared experiences of beauty a form of communication?” as additional predictor.**

| Random effects | | | | | |
| --- | --- | --- | --- | --- | --- |
|  | Variance | *SD* |  |  |  |
| Participant | 0.11 | 0.33 |  |  |  |
| Image | 0.22 | 0.47 |  |  |  |
| Fixed effects | | | | | |
|  | Estimate | *SE* | *df* | *t* | *p* |
| Intercept | -0.54 | 0.75 | 559.5 | -0.72 | 0.474 |
| Communication | 0.15 | 0.12 | 577.1 | 1.25 | 0.213 |
| Pleasure | 0.28 | 0.18 | 831.7 | 1.51 | 0.131 |
| Wish to continue | 0.16 | 0.15 | 848.4 | 1.10 | 0.271 |
| **Feeling alive** | **0.31** | **0.15** | **848.6** | **2.08** | **0.038** |
| **Universality** | **0.61** | **0.14** | **759.8** | **4.32** | **<0.001** |
| Number of felt connections | -0.01 | 0.17 | 800.8 | -0.04 | 0.971 |
| Longing | -0.03 | 0.14 | 712.2 | -0.24 | 0.814 |
| Feeling free of desire | 0.00 | 0.15 | 786.4 | 0.01 | 0.995 |
| Mind-wandering | -0.07 | 0.14 | 804.2 | -0.54 | 0.591 |
| Surprise | 0.01 | 0.13 | 757.4 | 0.07 | 0.944 |
| Wanting to understand more | -0.18 | 0.15 | 811.6 | -1.18 | 0.239 |
| Telling a story | 0.03 | 0.12 | 845.9 | 0.24 | 0.810 |
| Communication × pleasure | -0.03 | 0.03 | 823.7 | -0.81 | 0.416 |
| Communication × Wish to continue | 0.00 | 0.03 | 847.3 | 0.11 | 0.911 |
| **Communication × Feeling alive** | **-0.05** | **0.03** | **848.1** | **-2.04** | **0.041** |
| **Communication × Universality** | **-0.06** | **0.02** | **742.2** | **-2.42** | **0.016** |
| Communication × Number of felt connections | 0.01 | 0.03 | 807.6 | 0.47 | 0.642 |
| Communication × Longing | 0.01 | 0.02 | 723.0 | 0.53 | 0.599 |
| Communication × Feeling free of desire | 0.02 | 0.03 | 798.6 | 0.68 | 0.496 |
| Communication × Mind-wandering | 0.03 | 0.02 | 811.9 | 1.11 | 0.269 |
| Communication × Surprise | 0.00 | 0.02 | 747.6 | 0.11 | 0.911 |
| Communication × Wanting to understand more | 0.04 | 0.03 | 824.8 | 1.68 | 0.093 |
| Communication × Telling a story | -0.01 | 0.02 | 846.4 | -0.47 | 0.639 |

*Notes.* The reference level for image category was the beautiful art image category. Significant fixed effects and interactions are highlighted in bold. The model explained 69 % of the variance.

**Table S21. The linear-mixed effects model for Experiment 1b, including the general beauty attitude question “How much does mood affect the feeling of beauty?” as additional predictor.**

| Random effects | | | | | |
| --- | --- | --- | --- | --- | --- |
|  | Variance | *SD* |  |  |  |
| Participant | 0.08 | 0.28 |  |  |  |
| Image | 0.23 | 0.48 |  |  |  |
| Fixed effects | | | | | |
|  | Estimate | *SE* | *df* | *t* | *p* |
| Intercept | 1.03 | 0.99 | 581.6 | 1.04 | 0.299 |
| Mood | -0.10 | 0.16 | 586.8 | -0.66 | 0.513 |
| Pleasure | 0.13 | 0.19 | 815.4 | 0.69 | 0.492 |
| Wish to continue | -0.01 | 0.17 | 843.2 | -0.05 | 0.958 |
| Feeling alive | 0.23 | 0.16 | 846.6 | 1.46 | 0.145 |
| **Universality** | **0.79** | **0.14** | **765.4** | **5.55** | **<0.001** |
| Number of felt connections | -0.13 | 0.19 | 729.2 | -0.66 | 0.513 |
| Longing | -0.14 | 0.15 | 539.7 | -0.96 | 0.335 |
| Feeling free of desire | -0.02 | 0.15 | 841.6 | -0.12 | 0.907 |
| Mind-wandering | -0.09 | 0.15 | 627.6 | -0.63 | 0.526 |
| Surprise | -0.08 | 0.16 | 691.4 | -0.50 | 0.616 |
| Wanting to understand more | 0.16 | 0.15 | 846.4 | 1.04 | 0.298 |
| Telling a story | -0.12 | 0.13 | 851.7 | -0.95 | 0.342 |
| Mood × pleasure | 0.00 | 0.03 | 810.0 | 0.08 | 0.940 |
| Mood × Wish to continue | 0.03 | 0.03 | 841.2 | 1.18 | 0.238 |
| Mood × Feeling alive | -0.04 | 0.03 | 846.9 | -1.38 | 0.167 |
| **Mood × Universality** | **-0.09** | **0.02** | **754.3** | **-3.76** | **<0.001** |
| Mood × Number of felt connections | 0.03 | 0.03 | 738.2 | 0.99 | 0.324 |
| Mood × Longing | 0.03 | 0.02 | 533.9 | 1.31 | 0.192 |
| Mood × Feeling free of desire | 0.02 | 0.02 | 840.2 | 0.77 | 0.439 |
| Mood × Mind-wandering | 0.03 | 0.02 | 646.2 | 1.18 | 0.238 |
| Mood × Surprise | 0.02 | 0.03 | 681.5 | 0.67 | 0.503 |
| Mood × Wanting to understand more | -0.02 | 0.02 | 848.1 | -0.65 | 0.515 |
| Mood × Telling a story | 0.02 | 0.02 | 849.6 | 0.78 | 0.438 |

*Notes.* The reference level for image category was the beautiful art image category. Significant fixed effects and interactions are highlighted in bold. The model explained 68 % of the variance.

**Table S22. The linear-mixed effects model for Experiment 1b, including the general beauty attitude question “Can you name an object that everyone finds beautiful?” as additional predictor.**

| Random effects | | | | | |
| --- | --- | --- | --- | --- | --- |
|  | Variance | *SD* |  |  |  |
| Participant | 0.10 | 0.31 |  |  |  |
| Image | 0.20 | 0.45 |  |  |  |
| Fixed effects | | | | | |
|  | Estimate | *SE* | *df* | *t* | *p* |
| Intercept | 0.48 | 0.28 | 102.6 | 1.68 | 0.096 |
| Universally beautiful object (UBO) | -0.23 | 0.31 | 598.1 | -0.74 | 0.458 |
| **Pleasure** | **0.11** | **0.05** | **770.0** | **2.30** | **0.022** |
| Wish to continue | 0.06 | 0.05 | 841.5 | 1.30 | 0.193 |
| Feeling alive | 0.02 | 0.04 | 856.9 | 0.48 | 0.629 |
| **Universality** | **0.40** | **0.05** | **811.7** | **8.62** | **<0.001** |
| Number of felt connections | 0.08 | 0.05 | 757.4 | 1.63 | 0.104 |
| Longing | 0.01 | 0.04 | 697.6 | 0.24 | 0.808 |
| **Feeling free of desire** | **0.14** | **0.04** | **843.4** | **3.22** | **0.001** |
| Mind-wandering | 0.01 | 0.04 | 726.9 | 0.17 | 0.866 |
| Surprise | 0.06 | 0.04 | 696.9 | 1.64 | 0.103 |
| Wanting to understand more | 0.09 | 0.04 | 828.7 | 2.15 | 0.032 |
| Telling a story | -0.07 | 0.04 | 857.9 | -1.79 | 0.074 |
| UBO × pleasure | 0.07 | 0.07 | 786.0 | 1.05 | 0.293 |
| **UBO × Wish to continue** | **0.19** | **0.06** | **844.7** | **3.05** | **0.002** |
| UBO × Feeling alive | 0.01 | 0.06 | 847.8 | 0.10 | 0.918 |
| **UBO × Universality** | **-0.21** | **0.06** | **761.6** | **-3.69** | **<0.001** |
| UBO × Number of felt connections | -0.07 | 0.07 | 764.4 | -1.02 | 0.309 |
| UBO × Longing | 0.04 | 0.06 | 703.7 | 0.70 | 0.487 |
| UBO × Feeling free of desire | -0.08 | 0.05 | 843.3 | -1.52 | 0.130 |
| **UBO × Mind-wandering** | **0.13** | **0.05** | **751.2** | **2.47** | **0.014** |
| UBO × Surprise | -0.09 | 0.05 | 713.2 | -1.82 | 0.069 |
| UBO × Wanting to understand more | -0.04 | 0.06 | 826.5 | -0.70 | 0.484 |
| **UBO × Telling a story** | **0.09** | **0.05** | **847.4** | **1.98** | **0.048** |

*Notes.* The reference level for universal beauty was “yes”. Significant fixed effects and interactions are highlighted in bold. The model explained 69% of the variance.

# Combined data from Experiment 1a and 1b: additional analyses

## Model comparisons

We performed model comparisons using 10-fold cross-validation with the R package *cvms*. In addition, we also obtained goodness of fit measures for all models using the R package *performance*. Since the data from Experiment 1 contained one participant who did not wish to disclose their gender, we compared all models but the one including gender using the full data set and ran additional model comparisons that included the gender-interaction model excluding data of the participant without disclosed gender.

The 10-fold cross-validation results for the full data set are shown in **Table S23**, the ones including the gender-interaction in **Table S24**. Results from model fitting without cross-validation are shown in **Tables S25-26**.

**Table S23. 10-fold cross-validation results for Experiment 1, excluding the gender-interaction model.**

| Model | Statistic | | | |
| --- | --- | --- | --- | --- |
|  | AIC | BIC | Δ BIC | RMSE |
| Fixed effects, no interaction | 6898 | 6985 |  | 1.02 |
| Interaction: stimulus modality | 6829 | 6985 | 0 | 1.02 |
| Interaction: age | 6888 | 7044 | 59 | 1.03 |
| Random effects only | 8718 | 8741 | 1756 | 1.6 |
| Interaction: stimulus type* | Model did not converge | | | |

*Notes*. All values represent averages across the 10 folds. *in contrast to stimulus modality, stimulus type distinguished between different image categories, too.

**Table S24. Model-fitting results for Experiment 1, excluding the gender-interaction model.**

| Model | Statistic | | | | |
| --- | --- | --- | --- | --- | --- |
|  | AIC | BIC | Δ BIC | RMSE | R^2^ |
| Fixed effects, no interaction | 7735.66 | 7823.98 |  | 0.96 | 0.72 |
| Interaction: stimulus modality | 7718.49 | 7877.45 | 53.47 | 0.95 | 0.74 |
| Interaction: stimulus type* | 7794.86 | 8095.13 | 271.15 | 0.94 | 0.75 |
| Interaction: age | 7860.18 | 8019.15 | 195.17 | 0.95 | 0.72 |
| Random effects only | 9680.71 | 9704.26 | 1880.28 | 1.33 | 0.48 |

*Notes*: *in contrast to stimulus modality, stimulus type distinguished between different image categories, too.

**Table S25. 10-fold cross-validation results for Experiment 1, including the gender-interaction model and therefore based on a reduced data set.**

| Model | Statistic | | | |
| --- | --- | --- | --- | --- |
|  | AIC | BIC | Δ BIC | RMSE |
| Interaction: stimulus modality | 6717 | 6873 |  | 1.02 |
| Fixed effects, no interaction | 6788 | 6875 | 2 | 1.02 |
| Interaction: age | 6779 | 6934 | 59 | 1.02 |
| Interaction: gender | 6788 | 6944 | 10 | 1.02 |
| Random effects only | 8588 | 8611 | 1667 | 1.59 |
| Interaction: stimulus type* | Model did not converge | | | |

*Notes*. All values represent averages across the 10 folds. *in contrast to stimulus modality, stimulus type distinguished between different image categories, too.

**Table S26. Model-fitting results for Experiment 1, including the gender-interaction model and therefore based on a reduced data set.**

| Model | Statistic | | | | |
| --- | --- | --- | --- | --- | --- |
|  | AIC | BIC | Δ BIC | RMSE | R^2^ |
| Fixed effects, no interaction | 7613.58 | 7701.69 |  | 0.95 | 0.72 |
| Interaction: stimulus modality | 7594.02 | 7752.62 | 50.93 | 0.94 | 0.74 |
| Interaction: gender | 7674.58 | 7833.18 | 131.49 | 0.95 | 0.72 |
| Interaction: stimulus type* | 7674.22 | 7973.79 | 272.1 | 0.93 | 0.76 |
| Interaction: age | 7737.76 | 7896.36 | 194.67 | 0.95 | 0.72 |
| Random effects only | 9535.8 | 9559.29 | 1857.6 | 1.33 | 0.49 |

*Notes*: *in contrast to stimulus modality, stimulus type distinguished between different image categories, too.

## Additional linear mixed effects models tested

The model explaining the highest proportion of variance, 75%, is described in the main article. **Tables S27-S29** list the results of all remaining tested models.

**Table S27. The linear-mixed effects model for Experiment 1 (combined data) without interactions.**

| Random effects | | | | | |
| --- | --- | --- | --- | --- | --- |
|  | Variance | *SD* |  |  |  |
| Participant | 0.07 | 0.26 |  |  |  |
| Image | 0.13 | 0.36 |  |  |  |
| Fixed effects | | | | | |
|  | Estimate | *SE* | *df* | *t* | *p* |
| **Intercept** | **0.34** | **0.12** | **65.1** | **2.93** | **0.005** |
| **Pleasure** | **0.21** | **0.02** | **2583.9** | **11.04** | **<0.001** |
| **Wish to continue** | **0.20** | **0.02** | **2621.6** | **11.27** | **<0.001** |
| **Feeling alive** | **0.08** | **0.02** | **2644.8** | **4.30** | **<0.001** |
| **Universality** | **0.23** | **0.02** | **2212.1** | **12.23** | **<0.001** |
| **Number of felt connections** | **0.06** | **0.02** | **2393.3** | **3.81** | **<0.001** |
| **Longing** | **0.04** | **0.02** | **2133.5** | **2.38** | **0.017** |
| **Feeling free of desire** | **0.07** | **0.02** | **2443.7** | **4.25** | **<0.001** |
| **Mind-wandering** | **0.05** | **0.02** | **2439.5** | **3.42** | **0.001** |
| Surprise | -0.02 | 0.01 | 1882.0 | -1.13 | 0.260 |
| **Wanting to understand more** | **0.04** | **0.02** | **2566.6** | **2.76** | **0.006** |
| Telling a story | -0.01 | 0.01 | 2392.7 | -0.73 | 0.466 |

*Notes.* Significant fixed effects and interactions are highlighted in bold. The model explained 72 % of the variance.

**Table S28. The linear-mixed effects model for Experiment 1 (combined data) including gender as additional factor.**

| Random effects | | | | | |
| --- | --- | --- | --- | --- | --- |
|  | Variance | *SD* |  |  |  |
| Participant | 0.07 | 0.26 |  |  |  |
| Image | 0.12 | 0.35 |  |  |  |
| Fixed effects | | | | | |
|  | Estimate | *SE* | *df* | *t* | *p* |
| Intercept | 0.32 | 0.13 | 104.0 | 2.50 | 0.014 |
| Gender | 0.01 | 0.16 | 1068.0 | 0.08 | 0.935 |
| **Pleasure** | **0.18** | **0.02** | **2572.0** | **7.64** | **<0.001** |
| **Wish to continue** | **0.19** | **0.02** | **2575.0** | **8.15** | **<0.001** |
| **Feeling alive** | **0.12** | **0.02** | **2603.0** | **5.40** | **<0.001** |
| **Universality** | **0.22** | **0.02** | **2247.0** | **9.66** | **<0.001** |
| **Number of felt connections** | **0.08** | **0.02** | **2354.0** | **3.91** | **<0.001** |
| Longing | 0.03 | 0.02 | 1929.0 | 1.40 | 0.163 |
| **Feeling free of desire** | **0.06** | **0.02** | **2375.0** | **3.16** | **0.002** |
| **Mind-wandering** | **0.07** | **0.02** | **2490.0** | **3.52** | **<0.001** |
| Surprise | -0.02 | 0.02 | 1749.0 | -1.28 | 0.202 |
| Wanting to understand more | 0.02 | 0.02 | 2493.0 | 1.23 | 0.219 |
| Telling a story | -0.01 | 0.02 | 2491.0 | -0.34 | 0.737 |
| Gender × pleasure | 0.06 | 0.04 | 2487.0 | 1.52 | 0.128 |
| Gender × Wish to continue | 0.05 | 0.04 | 2545.0 | 1.36 | 0.173 |
| **Gender × Feeling alive** | **-0.11** | **0.04** | **2582.0** | **-3.04** | **0.002** |
| Gender × Universality | 0.02 | 0.03 | 2280.0 | 0.51 | 0.609 |
| Gender × Number of felt connections | -0.05 | 0.03 | 2295.0 | -1.60 | 0.111 |
| Gender × Longing | 0.02 | 0.03 | 2137.0 | 0.58 | 0.561 |
| Gender × Feeling free of desire | 0.01 | 0.03 | 2381.0 | 0.30 | 0.762 |
| Gender × Mind-wandering | -0.03 | 0.03 | 2321.0 | -1.01 | 0.315 |
| Gender × Surprise | 0.02 | 0.03 | 1844.0 | 0.83 | 0.410 |
| Gender × Wanting to understand more | 0.04 | 0.03 | 2504.0 | 1.45 | 0.147 |
| Gender × Telling a story | -0.01 | 0.03 | 2523.0 | -0.51 | 0.611 |

*Notes.* The reference level for gender was male. Significant fixed effects and interactions are highlighted in bold. The model explained 72% of the variance.

**Table S29. The linear-mixed effects model for Experiment 1 (combined data) including age as additional predictor.**

| Random effects | | | | | |
| --- | --- | --- | --- | --- | --- |
|  | Variance | *SD* |  |  |  |
| Participant | 0.06 | 0.25 |  |  |  |
| Image | 0.12 | 0.35 |  |  |  |
| Fixed effects | | | | | |
|  | Estimate | *SE* | *df* | *t* | *p* |
| Intercept | -0.15 | 0.29 | 670.1 | -0.53 | 0.600 |
| Age | 0.01 | 0.01 | 709.8 | 1.80 | 0.072 |
| **Pleasure** | **0.31** | **0.07** | **2602.0** | **4.59** | **<0.001** |
| **Wish to continue** | **0.31** | **0.06** | **2626.0** | **4.83** | **<0.001** |
| Feeling alive | 0.05 | 0.06 | 2601.0 | 0.83 | 0.408 |
| **Universality** | **0.26** | **0.06** | **2283.0** | **4.09** | **<0.001** |
| Number of felt connections | -0.08 | 0.06 | 2235.0 | -1.42 | 0.156 |
| **Longing** | **0.12** | **0.06** | **2147.0** | **2.08** | **0.038** |
| Feeling free of desire | 0.01 | 0.06 | 2344.0 | 0.16 | 0.875 |
| Mind-wandering | 0.10 | 0.06 | 2543.0 | 1.81 | 0.071 |
| Surprise | -0.08 | 0.05 | 1541.0 | -1.62 | 0.105 |
| Wanting to understand more | 0.08 | 0.05 | 2513.0 | 1.50 | 0.134 |
| Telling a story | -0.09 | 0.05 | 2610.0 | -1.85 | 0.065 |
| Age × pleasure | 0.00 | 0.00 | 2602.0 | -1.64 | 0.101 |
| Age × Wish to continue | 0.00 | 0.00 | 2625.0 | -1.66 | 0.096 |
| Age × Feeling alive | 0.00 | 0.00 | 2608.0 | 0.35 | 0.726 |
| Age × Universality | 0.00 | 0.00 | 2210.0 | -0.62 | 0.534 |
| **Age × Number of felt connections** | **0.00** | **0.00** | **2302.0** | **2.62** | **0.009** |
| Age × Longing | 0.00 | 0.00 | 2136.0 | -1.46 | 0.143 |
| Age × Feeling free of desire | 0.00 | 0.00 | 2346.0 | 1.01 | 0.314 |
| Age × Mind-wandering | 0.00 | 0.00 | 2571.0 | -0.89 | 0.372 |
| Age × Surprise | 0.00 | 0.00 | 1485.0 | 1.47 | 0.143 |
| Age × Wanting to understand more | 0.00 | 0.00 | 2551.0 | -0.65 | 0.517 |
| Age × Telling a story | 0.00 | 0.00 | 2622.0 | 1.75 | 0.080 |

*Notes.* Significant fixed effects and interactions are highlighted in bold. The model explained 72 % of the variance.

## Additional cumulative link mixed models tested

To verify that the results of the linear mixed effects models were not distorted by the assumption that ratings are interval-scaled, we re-ran the main analyses with cumulative link mixed models that treated all ratings as ordinal variables. We used the *clmm* function of the R package *ordinal* to implement these models. Based on BIC, the model predicting beauty ratings based on a combination of other ratings without interactions fit the data best (see **Table S30** for the detailed model estimates and **Table S31** for BIC comparison).

**Table S30. The best-fitting CLM model for Experiment 1.**

| Random effects | | | | | | |
| --- | --- | --- | --- | --- | --- | --- |
|  | Variance | *SD* | |  | |  |
| Participant | 0.27 | 0.51 | |  | |  |
| Image | 0.41 | 0.64 | |  | |  |
|  | Estimate | *SE* | | *z* | | *p* |
| **Pleasure** | **2.39** | **0.22** | | **11.12** | | **<0.001** |
| **Pleasure (cubic)** | **0.44** | **0.13** | | **3.37** | | **0.001** |
| **Wish to continue** | **1.78** | **0.20** | | **8.87** | | **<0.001** |
| **Wish to continue (quadratic)** | **0.39** | **0.15** | | **2.68** | | **0.007** |
| **Wish to continue (cubic)** | **0.33** | **0.12** | | **2.69** | | **0.007** |
| **Feeling alive** | **0.92** | **0.20** | | **4.58** | | **<0.001** |
| **Feeling alive (quadratic)** | **-0.39** | **0.15** | | **-2.53** | | **0.012** |
| **Universality** | **2.46** | **0.21** | | **11.74** | | **<0.001** |
| **Number of felt connections** | **0.65** | **0.20** | | **3.35** | | **0.001** |
| **Longing** | **0.60** | **0.19** | | **3.22** | | **0.001** |
| **Longing (cubic)** | **0.40** | **0.13** | | **3.02** | | **0.003** |
| **Feeling free of desire** | **0.81** | **0.18** | | **4.56** | | **<0.001** |
| **Mind-wandering** | **0.59** | **0.18** | | **3.36** | | **0.001** |
| **Mind-wandering (^4)** | **0.25** | **0.12** | | **2.18** | | **0.030** |
| Surprise | 0.21 | 0.19 | | 1.10 | | 0.273 |
| **Surprise (quadratic)** | **0.62** | **0.18** | | **3.50** | | **<0.001** |
| **Surprise (^4)** | **0.45** | **0.13** | | **3.53** | | **<0.001** |
| **Wanting to understand more** | **0.61** | **0.17** | | **3.47** | | **0.001** |
| **Wanting to understand more (quadratic)** | **0.44** | **0.14** | | **3.10** | | **0.002** |
| **Wanting to understand more (cubic)** | **0.32** | **0.12** | | **2.70** | | **0.007** |
| Telling a story | -0.16 | 0.17 | | -0.94 | | 0.349 |
| **Telling a story (^6)** | **0.22** | **0.10** | | **2.20** | | **0.028** |
| Threshold coefficients | | | | | | |
|  | Estimate | | *SE* | | *z* | |
| 1\|2 | -4.50 | | 0.20 | | -22.59 | |
| 2\|3 | -2.81 | | 0.18 | | -15.45 | |
| 3\|4 | -1.36 | | 0.18 | | -7.77 | |
| 4\|5 | 0.11 | | 0.17 | | 0.64 | |
| 5\|6 | 1.89 | | 0.18 | | 10.64 | |
| 6\|7 | 4.29 | | 0.19 | | 22.42 | |

*Notes.* All fixed effects are linear effects unless otherwise noted. Significant fixed effects and interactions are highlighted in bold. Non-significant non-linear effects are not displayed for readability.

**Table S31. BIC scores for CLMMs applied to data from Experiment 1 treating all ratings as ordinal variables.**

| Model | Statistic | |
| --- | --- | --- |
|  | BIC | Δ BIC |
| Fixed effects, no interaction | 7097 |  |
| Interaction: gender | 7437 | 339 |
| Interaction: age | 7574 | 477 |
| Interaction: stimulus modality | 8312 | 1215 |
| Random effects only | 8724 | 1627 |

## Cluster analyses

Apart from these general patterns, we also examined whether distinct groups of participants exhibited different correlational patterns between beauty and the other ratings. To do so, we performed k-means cluster analyses on the correlation coefficients for each other rating with beauty ratings per participant. We used the R package *NbClust* to evaluate the outcomes k-means cluster analyses. Participants with missing values (*N* = 16) were removed from these analyses. Of the 24 considered indices, 12 proposed that two clusters fit the pattern of correlations best, whereas fewer than nine proposed a higher number of clusters each. We therefore assessed possible individual differences based to participant assignments to one out of two clusters.

As illustrated in **Figure S2**, the pattern of correlations did not substantially differ between the two clusters. Instead, they differed in terms of the overall correlation strength with medium to high correlations in the first cluster (*N* = 112) and small to negligible correlations in the second cluster (*N* = 64). The two clusters did not differ in terms of gender, age, or their answers regarding any of the questions posed at the end of the survey, all *p* ≥ 0.119 according to two-sided Chi-Square and Kolmogorov-Smirnov tests.


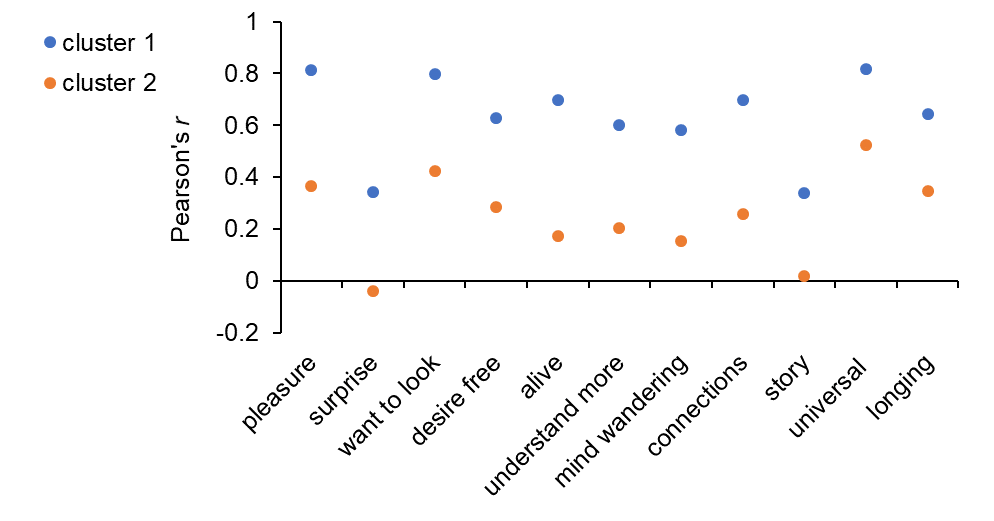

*Figure S2.* Average Pearson’s correlation coefficient between beauty and other rating dimensions listed on the horizontal axis per cluster (blue dots = cluster 1; orange dots = cluster 2).

# Experiment 2a &2b: Beauty memories (USA)

## Experiment 2a: text analysis.

For our text analysis, we first looked at the most frequently occurring words, excluding stop words and punctuation. Unsurprisingly, the most commonly used word was “beautiful” (*n* = 69), followed by “experience” (*n* = 35), and “could” (*n* = 28). Then came “like” which was just as frequently mentioned as “beauty” (*n* = 27). Predominantly, people were describing what they visually experienced, with both “see” (*n* = 26) and “saw” (*n* = 20) being among the 12 most frequent words. The remaining top-12 mentioned words were related to memory recall (“one”, “time”, “went”, “remember”).

In addition to the most frequent individual words we also looked at the most frequent word combinations, i.e., bigrams. Again, the most frequent reflected the task (“beauty experience” *n* = 9). The next most common “could see” (*n* = 8) confirms the dominance of visual experiences. Third, participants seemed to frequently recall special “first time” events (*n* = 7).

Going beyond the count of word frequencies, we used the *empath client* to analyze which lexical categories are overrepresented in our beauty descriptions compared to a standard text corpus. We find that the top ten lexical categories in the beauty memory descriptions were, in order: beauty, attractive, feminine, weather, beach, vacation, children, family, love, and water.

## Experiment 2b: additional demographic information.

Of the 89 participants whose date we analyzed, 43 held a college degree, 23 had some college education, 12 had a graduate degree, and 11 had High School education. 78 had no philosophy education at all, 9 attended some courses, and 2 obtained a philosophy degree. Similarly, 75 had no art education, 13 some, and one held a degree in art or related field. Most (59) were white, 13 black, 6 East Asian, 5 Hispanic, 3 South Asian, and one each identified as American Indian, Hawaiian, and multiracial. In terms of religion, 37 were Christian, 17 agnostic, 15 atheist, 4 Jewish, 2 Buddhist, and one each was Hindu and Noahidist. The median response to political affiliation was “slight liberal”, with a total of 51 participants placing themselves as liberal, 14 as “neutral”, and 24 as somewhere on the conservative side. The gross household income of those participants who did disclose it (2 did not), was below UDS 25,000 for 19, below USD 50,000 for 27, below USD 70,000 for 22, below USD 100,000 for 11, and 8 reported more than that.

## Experiment 2b: text analysis.

The text analysis of the second US-American sample’s memory descriptions revealed a highly similar pattern to the one obtained for the first sample. The most commonly used word was, again, “beautiful” (*n* = 65), followed by “beauty” (*n* = 28), and “felt” (*n* = 27). Then came “time” which was just as frequently mentioned as “day” (*n* = 27). “Like” was frequently used, too (*n* = 25). People used words related to visual nature experiences “see” (*n* = 19), “nature” (*n* = 20), and “sun” (*n* = 18), being among the most frequently used words. This underlines the results obtained so far and is in line with people’s general beliefs about beauty (see below *People’s explicit beliefs about beauty* in the main article). the most frequently occurring bigrams further confirm the predominance of beauty, experiential, and nature themes: “first time” (*n* =11), “years ago” (*n* =8), “beautiful experience” (*n* = 5), “ever seen” (*n* = 5), “looked like”, and “sun shining” (*n* = 4). Of note, as in the first US sample and the UK, US-Americans frequently mentioned “first time” experiences, a pattern that we did not find in India.

The *empath* analyses on the second US-American sample’s memory descriptions brought up similar if not identical themes compared to our first sample. The top 10 were beauty, attractive, feminine, weather, beach, vacation, love, plant, positive emotion, and traveling.

## Experiment 2b: Perceptual task.

The circle size ratings were mostly unrelated to aesthetic judgments. Out of 16 ratings, one correlated slightly positive, longing, *r* = 0.21, 95% CI [0.00, 0.40], and one slightly negative, beauty, *r* = -0.25, [-0.43, -0.04], with the circle size difference rating. Therefore, general biases can again not explain the overall rating pattern.

## Comparison between Experiment 2a & 2b


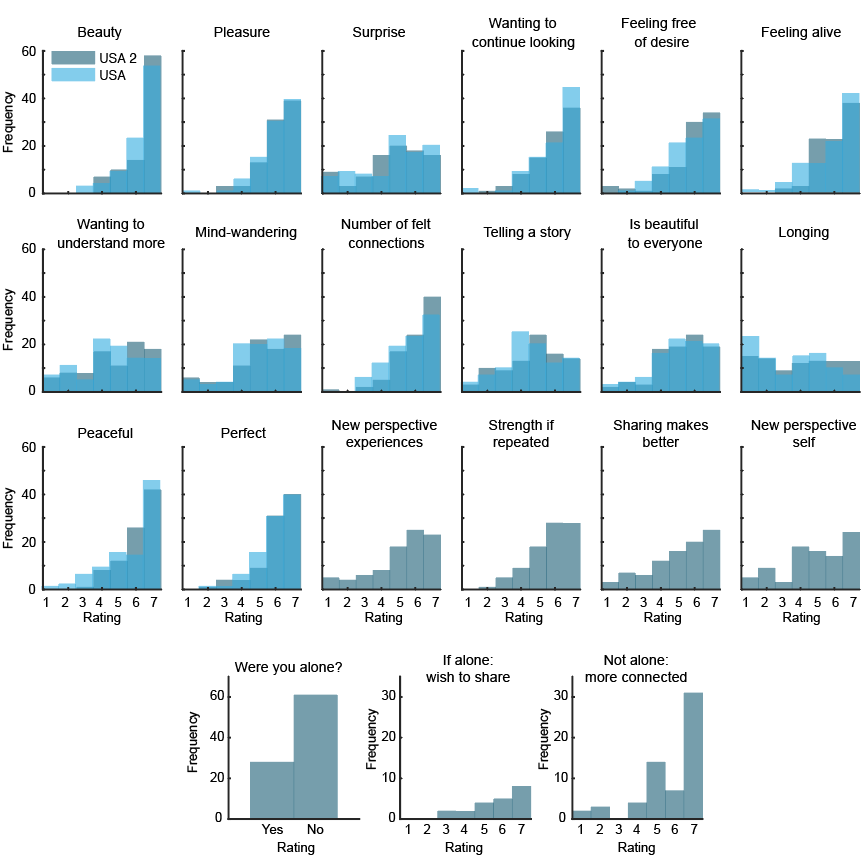


*Figure S3.* Histograms of ratings for beauty memories from the first US-American sample (light blue; Experiment 2a) and the second US-American sample (dark blue; Experiment 2b). Note that data for the last seven displayed ratings was collected from the second sample only. None of the average ratings differed between samples, all *p* ≥ 0.063.

# Additional comparisons between Experiment 1 and 2

We report comparisons between ratings correlated with beauty in the main manuscript. Here, we report differences along the remaining two dimensions. Ratings of surprise were higher for remembered beauty, *M* = 5.01, compared to immediate beauty, *M* = 3.84, *p* < 0.001. Ratings of how much the experience tells a story did not differ between remembered and immediate beauty, *p* = 0.966.

# Experiment 3a: Beauty memories in the UK

Participants from the US and the UK did not differ in their average ratings of beauty, Wish to continue the experience, feeling alive, and longing. The overall distribution of their responses was also similar, as illustrated in **Figure S4**. However, participants from the UK did give higher ratings on 10 out of 14 rating scales (see **Figure S4**).


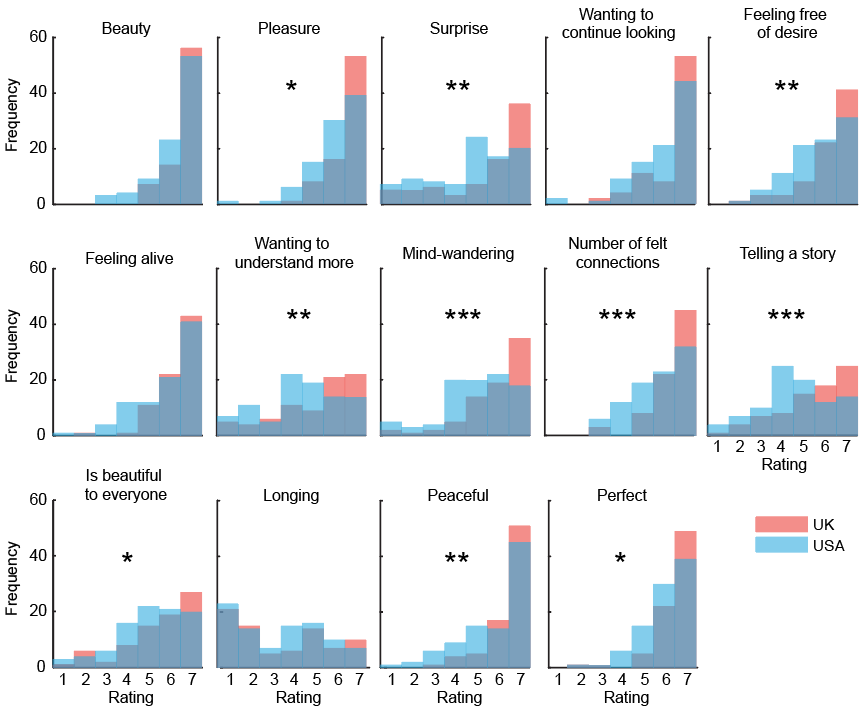


*Figure S4.* Histograms of ratings for beauty memories receiving maximum beauty ratings by US American (blue; Experiment 2a) and British participants (red; Experiment 3a). **p* < 0.05, ***p*< 0.01, ****p* < 0.001, according to two-sided t-tests.

## Text analysis.

The most frequently occurring words in the descriptions of beauty experiences of UK participants were: “day” (*n* =42), “beautiful” (*n* =38), “time” (*n* =31), “could” (*n* =30), “like” (*n* =28), “see” (*n* =26), “felt” (*n* =23), and “smell” (*n* = 21).

The most frequent bigrams for UK beauty memories were: “first time” (*n* =11), “could see” (*n* = 11), “felt like” (*n* = 4), “could feel” (*n* = 4), “summers day” (*n* = 3), and “could hear”, (*n* = 3). Thus, as opposed to US participants, people in the UK were less likely to tag the memory as beauty or beautiful experience but they exhibited the same proclivity to describe how they felt and what they saw.

The lexical analyses with *empath* revealed a large overlap in the top ten sentiments between US and UK, namely: beauty, attractive, weather, beach, feminine, children, water, and vacation. The theme party (ranking 18 out of 195 total sentiments in the US) and friends (ranking 20 in the US) were in the top ten in the UK but not the US, whereas the themes family (ranking 15 in the UK) and love (ranking 16 in the UK) appeared there in the US but not the UK.

# Experiment 3b: Beauty memories in India

Participants from the US and the India did not differ in their average ratings of beauty, pleasure, feeling free of desire, and peacefulness. The overall distribution of their responses was also similar, as illustrated in **Figure S5**. However, participants from India did give higher ratings on 9 out of 14 rating scales (see **Figure S5**).


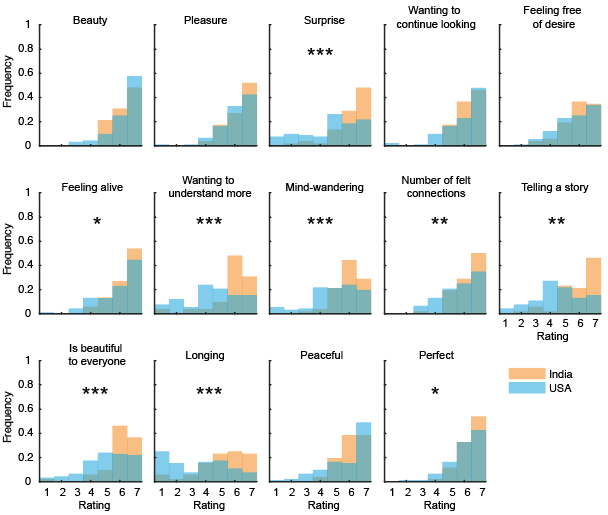


*Figure S5.* Histograms of ratings for beauty memories receiving maximum beauty ratings by US American (blue; Experiment 2a) and Indian participants (orange; Experiment 3b). **p* < 0.05, ***p*< 0.01, ****p* < 0.001 according to two-sided t-tests.

## Perceptual task.

The additional perception task of rating the magnitude of size difference between two circles served as an independent assessment of biases in the rating scale use. Participants had no difficulty judging which circle was bigger (50 out of 52 participants answered correct). Only one out of 14 aesthetic ratings correlated with the circle size rating, i.e., surprise, *r* = 0.50, 95% CI [0.26, 0.68]). Thus, participants’ ratings cannot be attributed to a generally biased use of the rating scales.

## Text analysis.

The most frequently occurring words in the descriptions of beauty experiences of participants in India were: “beautiful” (*n* =33), “felt” (*n* =23), “feel” (*n* =21), “day” (*n* =21), “beauty” (*n* =21), “time” (*n* =21), “like” (*n* = 20), and “experience” (*n* = 20).

The most frequent bigrams for UK beauty memories were: “beauty experience” (*n* =7), “years back” (*n* =6), “felt like” (*n* = 5), “beautiful experience” (*n* = 5), “back went” (*n* = 3), “beauty life” (*n* = 3), “years old” (*n* = 3), “like heaven” (*n* = 3), and “one day”, (*n* = 3).

The lexical analyses with *empath* revealed a considerable overlap in the top ten sentiments between US and India, namely: beauty, attractive, feminine, and love. The theme positive emotion (ranking 24 out of 195 total sentiments in the US), optimism (ranking 32 in the US), celebration (ranking 28 in the US), and contentment (ranking 48 in the US) were in the top ten in India but not the US. In contrast, the themes weather (ranking 18 in India), beach (ranking 36 in India), vacation (ranking 13 in India), children (ranking 19 in India), family (ranking 20 in India), and water (ranking 68 in India) appeared there in the US but not India.

# Experiment 5

## Experiment 5a: additional demographic information

Of the 101 participants, 45 had a college degree, 24 held a graduate degree, 21 had some college education, and 11 High School education. 81 had no philosophy education, 14 had attended some courses, and 6 held a philosophy degree. 78 had no art-related education, 16 had attended courses, and 7 held a degree in an art-related field. Of the 101, 10 participants indicated that they were professional artists. In terms of ethnicity, 81 participants were white, 8 black, 7 Hispanic, 3 East Asian, and one each South Asian and multiracial. On the liberal to conservative scale, the median response here was “neutral” (22 participants), while 47 identified as at least “slightly liberal”, and 32 as at least “slightly conservative”. The gross household income of those participants who did disclose it (one did not), was below UDS 25,000 for 16, below USD 50,000 for 34, below USD 70,000 for 24, below USD 100,000 for 12, and 14 reported more than that.

## Model comparisons

We employed the same model comparisons for Experiment 5 as for Experiment 1. In addition to considering various interaction terms, we also compared the performances of models with a linear versus squared complexity and excitement term. The results of the 10-fold cross-validation are displayed in **Table S32**, the results of the model fitting in **Table S33**.

**Table S32. 10-fold cross-validation results for Experiment 5.**

| Model | Statistic | | | |
| --- | --- | --- | --- | --- |
|  | AIC | BIC | Δ BIC | RMSE |
| Interaction: stimulus modality; squared* | 5795 | 5935 |  | 1.02 |
| Interaction: stimulus modality | 5798 | 5939 | 4 | 1.02 |
| Fixed effects, no interactions; squared* | 5863 | 5942 | 7 | 1.03 |
| Fixed effects, no interactions | 5872 | 5950 | 15 | 1.03 |
| Interaction: age | 5863 | 6004 | 69 | 1.03 |
| Interaction: gender | 5879 | 6019 | 84 | 1.04 |
| Interaction: stimulus type** | 5759 | 6023 | 88 | 1.02 |
| Interaction: stimulus type**; squared* | 5779 | 6043 | 108 | 1.02 |
| Random effects only | 7424 | 7446 | 1511 | 1.59 |

*Notes*. All values represent averages across the 10 folds. *the predictors that were re-coded as centered, squared values in these models were complexity and excitement; **in contrast to stimulus modality, stimulus type distinguished between different image categories, too.

**Table S33. Model-fitting results for Experiment 5.**

| Model | Statistic | | | | |
| --- | --- | --- | --- | --- | --- |
|  | AIC | BIC | Δ BIC | RMSE | R^2^ |
| Fixed effects, no interaction; squared* | 6579.99 | 6660.11 |  | 0.96 | 0.68 |
| Fixed effects, no interaction | 6585.93 | 6666.05 | 5.94 | 0.96 | 0.68 |
| Interaction: stimulus modality | 6552.48 | 6695.55 | 35.44 | 0.94 | 0.7 |
| Interaction: stimulus modality; squared* | 6555.08 | 6698.14 | 38.03 | 0.94 | 0.7 |
| Interaction: gender | 6646.12 | 6789.19 | 129.08 | 0.96 | 0.68 |
| Interaction: age | 6700.03 | 6843.1 | 182.99 | 0.95 | 0.68 |
| Interaction: stimulus type**; squared* | 6604.76 | 6873.73 | 213.62 | 0.93 | 0.72 |
| Interaction: stimulus type** | 6638.92 | 6907.89 | 247.78 | 0.93 | 0.72 |
| Random effects only | 8243.72 | 8266.61 | 1606.5 | 1.33 | 0.44 |

*Notes*: *the predictors that were re-coded as centered, squared values in these models were complexity and excitement; **in contrast to stimulus modality, stimulus type distinguished between different image categories, too.

## Additional cumulative link mixed models tested

As for Experiment 1, we verified that the results of the linear mixed effects models were not distorted by the assumption that ratings are interval-scaled by running the main analyses with cumulative link mixed models that treated all ratings as ordinal variables. Based on BIC, the model predicting beauty ratings based on a combination of other ratings without interactions fit the data best (see **Table S34** for the detailed model estimates and **Table S35** for BIC comparison). Note that, like for the standard linear-mixed effect model, the interaction of stimulus category and interest was significant when treating interest as an ordinal variable, Estimate = -1.46, *SE* = 0.73, *z* = -2.01, *p* = 0.0449, and the direction and magnitude of this interaction again indicated that interest and beauty did not correlate for songs. Also note that .

**Table S34. The best-fitting CLM model for Experiment 5.**

| Random effects | | | | | | |
| --- | --- | --- | --- | --- | --- | --- |
|  | Variance | *SD* | |  | |  |
| Participant | 0.19 | 0.43 | |  | |  |
| Image | 0.37 | 0.61 | |  | |  |
|  | Estimate | *SE* | | *z* | | *p* |
| **Pleasure** | **2.26** | **0.24** | | **9.28** | | **<0.0001** |
| **Pleasure^4** | **0.26** | **0.13** | | **2.03** | | **0.042** |
| **Pleasure^6** | **0.30** | **0.11** | | **2.61** | | **0.009** |
| Complexity | 0.30 | 0.19 | | 1.56 | | 0.119 |
| **Complexity (quadratic)** | **0.31** | **0.14** | | **2.20** | | **0.028** |
| Excitement | 0.13 | 0.21 | | 0.61 | | 0.542 |
| **Excitement (cubic)** | **0.27** | **0.14** | | **1.98** | | **0.047** |
| Learning | 0.18 | 0.19 | | 0.96 | | 0.339 |
| Understandable | 0.43 | 0.28 | | 1.54 | | 0.123 |
| **Harmony in variety** | **0.84** | **0.25** | | **3.42** | | **0.001** |
| **Harmony in variety ?(quadratic)** | **0.44** | **0.21** | | **2.09** | | **0.037** |
| **Meaningful** | **1.06** | **0.24** | | **4.41** | | **<0.0001** |
| **Exceeded expectations** | **1.23** | **0.23** | | **5.34** | | **<0.0001** |
| **Interest** | **1.41** | **0.26** | | **5.48** | | **<0.0001** |
| **Moved** | **2.04** | **0.25** | | **8.20** | | **<0.0001** |
| **Moved (quadratic)** | **-0.39** | **0.19** | | **-2.08** | | **0.037** |
| **Moved (cubic)** | **0.48** | **0.15** | | **3.26** | | **0.001** |
| Threshold coefficients | | | | | | |
|  | Estimate | | *SE* | | *z* | |
| 1\|2 | -4.28 | | 0.21 | | -20.20 | |
| 2\|3 | -2.35 | | 0.18 | | -12.70 | |
| 3\|4 | -1.05 | | 0.18 | | -5.95 | |
| 4\|5 | 0.41 | | 0.18 | | 2.33 | |
| 5\|6 | 2.15 | | 0.18 | | 11.90 | |
| 6\|7 | 4.29 | | 0.19 | | 22.24 | |

*Notes.* All fixed effects are linear effects unless otherwise noted. Significant fixed effects and interactions are highlighted in bold. Non-significant non-linear effects are not displayed for readability.

**Table S35. BIC scores for CLMMs applied to data from Experiment 5 treating all ratings as ordinal variables.**

| Model | Statistic | |
| --- | --- | --- |
|  | BIC | Δ BIC |
| Fixed effects, no interaction | 6163.10 | - |
| Interaction: stimulus modality | 6490.68 | 327.58 |
| Interaction: gender | 6580.55 | 417.45 |
| Interaction: age | 6594.99 | 431.89 |
| Random effects only | 7463.23 | 1300.13 |

## Cluster analyses

We used the same cluster analyses as for Experiment 1. Participants with missing values (*N* = 21) were again removed from these analyses. Of the 24 considered indices, 12 proposed that two clusters fit the pattern of correlations best, whereas fewer than seven proposed a higher number of clusters each. We therefore assessed possible individual differences based to participant assignments to one out of two clusters.

As illustrated in **Figure S6**, and similar to the pattern observed in data from Experiment 1, clusters mostly differed in terms of the overall correlation strength. Again, correlations were medium to high in the first cluster (*N* = 123) and small to negligible correlations in the second cluster (*N* = 51). In contrast to Experiment 1, we did find further differences between these clusters. Participants in cluster 1 were older, *Mdn* = 37, than in cluster 2, *Mdn* = 29, *D* = 0.45, *p* < 0.001. Participants in cluster 2 were more likely to state that a universally beautiful object exists (72% yes responses) than participants of cluster 1 (46% yes responses). People in cluster 2 also tended to rate beauty as lying more in an object’s story than people in cluster 1, *Mdn* = 5 and *Mdn* = 4 on a scale from 1 = “entirely in the image” to 4 = “both to 7 = “entirely in the story the image tells you”. Otherwise, we found no differences between clusters, all *p* ≥ 0.159.

*Figure S6.* Average Pearson’s correlation coefficient between beauty and other rating dimensions listed on the horizontal axis per cluster (blue dots = cluster 1; orange dots = cluster 2).

# Experiment 6

## Additional demographic information

Of the 72 participants whose data we analyzed, 36 had a college degree, 12 held a graduate degree, 13 had some college education, and 11 High School education. 62 had no philosophy education, 9 had attended some courses, and one held a philosophy degree. 54 had no art-related education, 17 had attended courses, and 1 held a degree in an art-related field. In terms of ethnicity, 56 participants were white, 8 black, 5 Hispanic, and one each East Asian, Hawaiian, and multiracial. On the liberal to conservative scale, the median response here was “slightly liberal”, while 7 identified as at least “neutral”. A total of 38 participants identified themselves as somewhere on the liberal spectrum, and 27 as at least “slightly conservative”. The gross household income of those participants who did disclose it (one did not), was below UDS 25,000 for 10, below USD 50,000 for 30, below USD 70,000 for 19, below USD 100,000 for 6, and 6 reported more than that.

## Text analysis.

The most frequently occurring words in the descriptions of beauty experiences of participants in India were: “beautiful” (*n* =50), “time” (*n* =33), “day” (*n* =32), “beauty” (*n* =24), “remember” (*n* =20), “one” (*n* =19), “see” (*n* = 19), and “went” (*n* = 17). The most frequent bigrams for beauty memories in Experiment 6 were: “first time” (*n* =9), “could see” (*n* =5), and “made feel” (*n* = 4). The lexical analyses with *empath* revealed that the top 10 most prevalent themes were: beauty, attractive, feminine, family, children, wedding, positive emotion, weather, and party.

# Additional experiment: comparing beauty and joy memories

## Methods

*Participants.* Of the 100 recruited participants, 99 completed the survey. Based on the written memory descriptions provided, we excluded nine participants (7 men, 2 women) due to apparent non-compliance. Of the remaining 90 participants, 58 were male, 31 female, and one preferred not to disclose their gender. Their age ranged from 20 to 71 with a mean of 36.6 years (*SD* = 11.5).

*Procedures.* The procedures were identical to Experiment 3 except for replacing the word “beauty” with “joy”. However, participants still rated how much beauty they felt during the experience.

*Analyses.* We used the same analyses as for Experiment 3.

## Results

Average ratings for beauty versus joy memories differed in two aspects: Joy memories were accompanied by a greater feeling of being alive, *M* = 6.20, than beauty memories, *M* = 5.17, *p* = 0.001. Plus, people felt more strongly that their joy experience is joyful to everyone, *M* = 4.04, than they felt that their beauty experience is beautiful to everyone, *M* = 3.57, *p* = 0.013. Ratings of beauty and joy memories did not differ along any other tested dimension, all *p* ≥ 0.122.

The most frequently occurring words in the descriptions of joy experiences were related to task and recall: “time” (*n* = 58), “first” (*n* = 47), “day” (*n* = 38), “went” (*n* = 34), “felt” (*n* = 34), and “joy” (*n* = 34). Next came “happy” (*n* = 29), and “see” (*n* = 27), again showing that people mostly described a visual experience, just like they did for beauty. Notably, people also frequently mentioned “beautiful” (*n* = 22) when describing their experiences of joy.

The most frequent bigrams for joy memories were: “first time” (*n* = 23), “intense joy” (*n* = 9), “felt intense” (*n* = 7), followed by “best friend” (*n* = 6) and “daughter born” (*n* = 5). Thus, it seems, when people are asked about intense joy experiences, they recall special first-time events more so than when they think about a beauty experience. A more frequent connection with being with other people also seems apparent and was confirmed by the lexical analyses with *empath*. The top ten *empath client* categories for joy were: positive emotion, family, optimism, children, love, friends, party, celebration, childish, wedding.

Overall, beauty and joy experiences seem to share a large portion of their characteristics. This should not come as a surprise given that many joy experiences were also rated to be beautiful. Text analyses, however, revealed that different themes dominate people’s memories when asking for joy vs. pleasure. While beauty memories often occur in the context of nature, joy memories seem to be more social in nature and frequently involve family and friends.

# References

Armstrong, T., & Detweiler-Bedell, B. (2008). Beauty as an emotion: The exhilarating prospect of mastering a challenging world. *Review of general psychology*, *12*(4), 305-329.

Berlyne, D. E. (1971). *Aesthetics and Psychobiology.* New. York: Appleton-Century-Crofts.

Charrad, M., Ghazzali, N., Boiteau, V., & Niknafs, A. (2014). NbClust: An R Package for Determining the Relevant Number of Clusters in a Data Set. *Journal of Statistical Software, 61*(6), 1-36. URL http://www.jstatsoft.org/v61/i06/.

Christensen, R. H. B. (2019). ordinal - Regression Models for Ordinal Data. R package version 2019.12-10. <https://CRAN.R-project.org/package=ordinal>.

Denham, A. E. (Ed.) (2012) *Plato on Art and Beauty*. New York, NY: Palgrave MacMillan.

Diessner, R., Pohling, R., Stacy, S., & Güsewell, A. (2018). Trait appreciation of beauty: A story of love, transcendence, and inquiry. *Review of General Psychology*, *22*(4), 377-397.

Fechner, G. T. (1876). *Vorschule der Aesthetik* (Vol. 1). Breitkopf & Härtel.

Halliwell, S. (1986). *Aristotle’s Poetics*. Chapel Hill, NC: University of North Carolina Press.

Ishizu, T., & Zeki, S. (2011). Toward a brain-based theory of beauty. PloS one, 6(7).

Kivy, P. (1990). *Music alone: Philosophical reflections on the purely musical experience*. Cornell University Press.

Leder, H., Belke, B., Oeberst, A., & Augustin, D. (2004). A model of aesthetic appreciation and aesthetic judgments. *British journal of psychology*, *95*(4), 489-508.

Ludvig Renbo Olsen and Benjamin Hugh Zachariae (2021). cvms: Cross-Validation for Model Selection. R package version 1.2.1. <https://CRAN.R-project.org/package=cvms>

Menninghaus, W., Wagner, V., Kegel, V., Knoop, C. A., & Schlotz, W. (2019). Beauty, elegance, grace, and sexiness compared. *PLOS ONE*, *14*(6), e0218728.

Nuzzo, A. (2005). *Kant and the Unity of Reason.* West Lafayette, IN: Purdue University Press.

Nuzzo, A. (2006). Hegel’s ‘Aesthetics’ as Theory of Absolute Spirit. *Internationales Jahrbuch des Deutschen Idealismus/International Yearbook of German Idealism*, 4, 291-310

Redies, C. (2014). Beauty: neglected, but alive and kicking. *British Journal of Psychology*, *105*(4), 468-470.

Salimpoor, V. N., Zald, D. H., Zatorre, R. J., Dagher, A., & McIntosh, A. R. (2015). Predictions and the brain: how musical sounds become rewarding. *Trends in cognitive sciences*, *19*(2), 86-91.

Taylor, J. (2008). *Hume*. In: Hume on Beauty and Virtue. Radcliff, E. (Ed.). Oxford: Blackwell.

Vessel, Edward A., G. Gabrielle Starr, and Nava Rubin. "Art reaches within: aesthetic experience, the self and the default mode network." *Frontiers in Neuroscience* 7 (2013): 258.
